# Comparative Polygenic Analysis of Maximal Ethanol Accumulation Capacity and Tolerance to High Ethanol Levels of Cell Proliferation in Yeast

**DOI:** 10.1371/journal.pgen.1003548

**Published:** 2013-06-06

**Authors:** Thiago M. Pais, María R. Foulquié-Moreno, Georg Hubmann, Jorge Duitama, Steve Swinnen, Annelies Goovaerts, Yudi Yang, Françoise Dumortier, Johan M. Thevelein

**Affiliations:** 1 Laboratory of Molecular Cell Biology, Institute of Botany and Microbiology, KU Leuven, Leuven-Heverlee, Flanders, Belgium; 2 Department of Molecular Microbiology, VIB, Leuven-Heverlee, Flanders, Belgium; 3 Agrobiodiversity Research Area, International Center for Tropical Agriculture (CIAT), Cali, Colombia; Duke University, United States of America

## Abstract

The yeast *Saccharomyces cerevisiae* is able to accumulate ≥17% ethanol (v/v) by fermentation in the absence of cell proliferation. The genetic basis of this unique capacity is unknown. Up to now, all research has focused on tolerance of yeast cell proliferation to high ethanol levels. Comparison of maximal ethanol accumulation capacity and ethanol tolerance of cell proliferation in 68 yeast strains showed a poor correlation, but higher ethanol tolerance of cell proliferation clearly increased the likelihood of superior maximal ethanol accumulation capacity. We have applied pooled-segregant whole-genome sequence analysis to identify the polygenic basis of these two complex traits using segregants from a cross of a haploid derivative of the sake strain CBS1585 and the lab strain BY. From a total of 301 segregants, 22 superior segregants accumulating ≥17% ethanol in small-scale fermentations and 32 superior segregants growing in the presence of 18% ethanol, were separately pooled and sequenced. Plotting SNP variant frequency against chromosomal position revealed eleven and eight Quantitative Trait Loci (QTLs) for the two traits, respectively, and showed that the genetic basis of the two traits is partially different. Fine-mapping and Reciprocal Hemizygosity Analysis identified *ADE1*, *URA3,* and *KIN3*, encoding a protein kinase involved in DNA damage repair, as specific causative genes for maximal ethanol accumulation capacity. These genes, as well as the previously identified *MKT1* gene, were not linked in this genetic background to tolerance of cell proliferation to high ethanol levels. The superior *KIN3* allele contained two SNPs, which are absent in all yeast strains sequenced up to now. This work provides the first insight in the genetic basis of maximal ethanol accumulation capacity in yeast and reveals for the first time the importance of DNA damage repair in yeast ethanol tolerance.

## Author Summary

The yeast *Saccharomyces cerevisiae* is unique in being the most ethanol tolerant organism known. This property lies at the basis of its ecological competitiveness in sugar-rich ecological niches and its use for the production of alcoholic beverages and bioethanol, both of which involve accumulation of high levels of ethanol. Up to now, all research on yeast ethanol tolerance has focused on tolerance of cell proliferation to high ethanol levels. However, the most ecologically and industrially relevant aspect is the capacity of fermenting yeast cells to accumulate high ethanol levels in the absence of cell proliferation. Using QTL mapping by pooled-segregant whole-genome sequence analysis, we show that maximal ethanol accumulation capacity and tolerance of cell proliferation to high ethanol levels have a partially different genetic basis. We identified three specific genes responsible for high ethanol accumulation capacity, of which one gene encodes a protein kinase involved in DNA damage repair. Our work provides the first insight in the genetic basis of maximal ethanol accumulation capacity, shows that it involves different genetic elements compared to tolerance of cell proliferation to high ethanol levels, and reveals for the first time the importance of DNA damage repair in ethanol tolerance.

## Introduction

The capacity to produce high levels of ethanol is a very rare characteristic in nature. It is most prominent in the yeast *Saccharomyces cerevisiae*, which is able to accumulate in the absence of cell proliferation, ethanol concentrations in the medium of more than 17%, a level that kills virtually all competing microorganisms. As a result this property allows this yeast to outcompete all other microorganisms in environments rich enough in sugar to sustain the production of such high ethanol levels [1], [2]. Very few other microorganisms, e.g. the yeast *Dekkera bruxellensis*, have independently evolved a similar but less pronounced ethanol tolerance compared to *S. cerevisiae* [3]. The capacity to accumulate high ethanol levels lies at the basis of the production of nearly all alcoholic beverages as well as bioethanol in industrial fermentations by the yeast *S. cerevisiae*. Originally, all alcoholic beverages were produced with spontaneous fermentations in which *S. cerevisiae* gradually increases in abundance, in parallel with the increase in the ethanol level, to finally dominate the fermentation at the end.

The genetic basis of yeast ethanol tolerance has attracted much attention but until recently nearly all research was performed with laboratory yeast strains, which display much lower ethanol tolerance than the natural and industrial yeast strains. This research has pointed to properties like membrane lipid composition, chaperone protein expression and trehalose content, as major requirements for ethanol tolerance of laboratory strains [2], 4 but the role played by these factors in other genetic backgrounds and in establishing tolerance to very high ethanol levels has remained unknown. We have recently performed polygenic analysis of the high ethanol tolerance of a Brazilian bioethanol production strain VR1. This revealed the involvement of several genes previously never connected to ethanol tolerance and did not identify genes affecting properties classically considered to be required for ethanol tolerance in lab strains [5].

A second shortcoming of most previous studies is the assessment of ethanol tolerance solely by measuring growth on nutrient plates in the presence of increasing ethanol levels [2], [4]. This is a convenient assay, which allows hundreds of strains or segregants to be phenotyped simultaneously with little work and manpower. However, the real physiological and ecological relevance of ethanol tolerance in *S. cerevisiae* is its capacity to accumulate by fermentation high ethanol levels in the absence of cell proliferation. This generally happens in an environment with a large excess of sugar compared to other essential nutrients. As a result, a large part of the ethanol in a typical, natural or industrial, yeast fermentation is produced with stationary phase cells in the absence of any cell proliferation. The ethanol tolerance of the yeast under such conditions determines its maximal ethanol accumulation capacity, a specific property of high ecological and industrial importance. In industrial fermentations, a higher maximal ethanol accumulation capacity allows a better attenuation of the residual sugar and therefore results in a higher yield. A higher final ethanol titer reduces the distillation costs and also lowers the liquid volumes in the factory, which has multiple beneficial effects on costs of heating, cooling, pumping and transport of liquid residue. It also lowers microbial contamination and the higher ethanol tolerance of the yeast generally also enhances the rate of fermentation especially in the later stages of the fermentation process. Maximal ethanol accumulation capacity can only be determined in individual yeast fermentations, which are much more laborious to perform than growth tests on plates. In static industrial fermentations, maintenance of the yeast in suspension is due to the strong CO_2_ bubbling and this can only be mimicked in lab scale with a sufficient amount of cells in a sufficiently large volume.

The advent of high-throughput methods for genome sequencing has created a breakthrough also in the field of quantitative or complex trait analysis in yeast [6], [7]. The new methodology has allowed efficient QTL mapping of several complex traits [5], [8], [9] and reciprocal hemizygosity analysis [10] has facilitated identification of the causative genes. The efficiency of the new methodologies calls for new challenges to be addressed, such as comparison of the genetic basis of related complex properties. In addition, complex trait analysis in yeast has been applied up to now mainly to phenotypic properties that are easy to score in hundreds or even thousands of segregants [5], [8]–[16]. However, many phenotypic traits with high ecological or industrial relevance require more elaborate experimental protocols for assessment and it is not fully clear yet whether the low numbers of segregants that can be scored in these cases are adequate for genetic mapping with pooled-segregant whole-genome sequence analysis.

The aim of this work was to compare the genetic basis of the complex traits of maximal ethanol accumulation capacity and tolerance of cell proliferation to high ethanol levels. We show that both traits have a partially different genetic basis and we have identified for the first time specific genes involved in maximal ethanol accumulation capacity.

## Results

### Strain selection for maximal ethanol accumulation capacity

**Figure 1:**
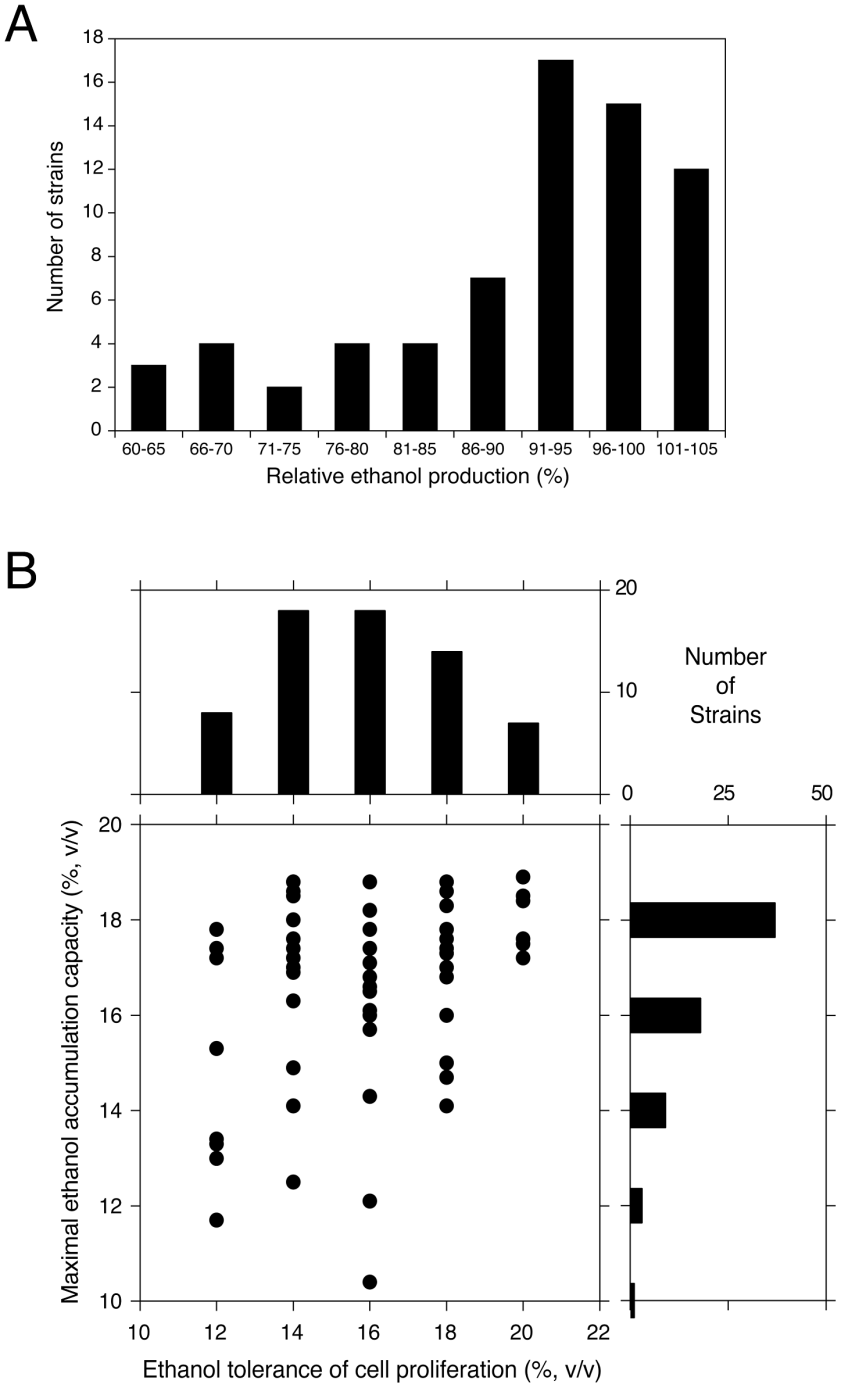
Maximal ethanol accumulation capacity and ethanol tolerance of cell proliferation in 68 different yeast strains. (A) Distribution of relative maximal ethanol production capacity of 68 different yeast strains compared to the wine strain V1116. The semi-static fermentations were performed in 250 mL of YP+33% glucose at 25°C. The V1116 strain produced 18.4% (±0.4%) (v/v) ethanol. (B) Ethanol tolerance of cell proliferation (X-axis) versus maximal ethanol accumulation capacity (Y-axis), expressed as maximal ethanol titer reached, in the 68 yeast strains. The highest ethanol concentration for which there was growth in all dilutions was taken as the maximal ethanol tolerance of cell proliferation. The possible correlation between the two traits was tested with a Spearman test, because of the non-normality of the ethanol accumulation trait. The (one-tailed) Spearman test indicated a weak correlation (90% confidence interval, P-value = 0.0984).

We have evaluated 68 different yeast strains in small-scale fermentations for maximal ethanol accumulation capacity under very high gravity (VHG) conditions [17], using 33% (w/v) glucose. The robust wine strain V1116 was used as reference in each series of fermentation experiments. Figure 1A shows the number of strains able to accumulate a certain maximal ethanol level expressed as percentage of the ethanol level accumulated by V1116 in the same experiment, which was 18.4±0.4% (v/v). There was no correlation between the final glycerol and ethanol levels produced but there was an inverse correlation between the final glycerol level and the ethanol yield. Table 1 shows the fermentation results for a number of representative strains ranked according to the maximal ethanol level produced in comparison with the reference V1116.

**Table 1:** Fermentation results for representative strains from the screen of 68 yeast strains.

Strains	Relative maximal ethanol accumulation (% compared to V1116)	Final ethanol titer (% v/v)	Glycerol titer (g/L)	Ethanol yield* (%)
CBS1585	103.4	18.8	10.9	88.4
CAT1	97.8	17.5	11.3	88.1
CBS6412	92.9	16.9	7.2	89.8
CBS2807	88.9	15.3	11.2	88.1
S288c	80.2	14.9	10.8	88.6
CBS1200	76.5	14.3	8.7	89.2
CBS382	74.7	14.1	10.8	88.4
CMBS33	66	12.5	10	88.7
BY4741	64.3	12.1	9.7	89.1

* Ethanol yield is expressed as percentage of the maximum theoretical ethanol yield (0.51 g ethanol/g glucose consumed).

High-gravity, semi-anaerobic, semi-static fermentations were carried out with 250 mL of YP+33% (w/v) glucose at 25°C.

The fermentation of the reference strain, V1116, took 9.4±1.1 days to complete. The ethanol productivity was 0.65 g.L^−1^.h^−1^ (or 0.83 g.L^−1^.h^−1^ when we omit the last two days where the fermentation had slowed down very much). The productivity was highest during the first three days (1.17 g.L^−1^.h^−1^). The yield was 0.446 g ethanol/g glucose (87.4%). There was 2.20±0.57% (w/v) glucose leftover. Glycerol production was 10.34±0.47 g/L. The final pH was 4.5±0.2 for all strains evaluated. The best ethanol producer was the sake strain, CBS1585, that accumulated 103.4% of the amount of ethanol accumulated by V1116. The relative ethanol production (% compared to V1116), the final ethanol % (v/v), the glycerol yield (g/L) and ethanol yield (% of maximum theoretical yield) for all 68 strains are listed in Table S1.

The laboratory strains BY4741 (*Mat* ***a*** *his3Δ1 leu2Δ0 ura3Δ0 met15Δ0*) and S288c (prototrophic) produced only 64% and 80%, respectively, of the ethanol level accumulated by V1116. This is in accordance with previous studies that showed the prototrophic laboratory strain (S288c) to be generally more stress tolerant than its auxotrophic counterpart (BY4741) [18], although this has not yet been documented for ethanol tolerance. The eight beer strains tested all produced less than 80% of the ethanol produced by V1116, in agreement with the relatively low ethanol levels generally present in beers. On the other hand, strains used for the production of bioethanol and sake were among the best for maximal ethanol accumulation, which fits with the high level of ethanol produced in these industrial fermentations [19], [20].

Cell viability at the end of the fermentation was lower than 10%, and usually only 1–5%, for all strains tested, except for Ethanol Red and CBS1585. The bioethanol production strain Ethanol Red retained 22.1%±4.1% viable cells and the sake strain, CBS1585, even 31.5%±5.1%. The latter strain also showed the highest ethanol accumulation among all strains evaluated. High ethanol production is a well-known trait of sake strains [21]. The high residual viability is remarkable in view of the 18–19% of ethanol accumulated. The ethanol level could be enhanced further by applying continuous stirring (200 rpm) and raising the glucose concentration to 35%. In this case, ethanol levels between 20 and 20.5% (v/v) were routinely obtained, with an absolute maximum of 20.9% (v/v). In six consecutive fermentations with the same cells under these conditions, 20.5% ethanol was accumulated in the first fermentation and 16.5–19.5% ethanol (v/v) in the subsequent fermentations, demonstrating the persistent viability of strain CBS1585 under high ethanol conditions.

We have compared the maximal ethanol accumulation capacity with the ethanol tolerance of cell proliferation in the 68 strains. The results are summarized in Figure 1B and all original data are provided in Table S1. The results show that most strains with a low ethanol tolerance of cell proliferation also displayed poor maximal ethanol accumulation and that none of these strains reached a final ethanol titer of more than 18% (v/v). Strains with a higher ethanol tolerance of cell proliferation tended to produce higher maximal ethanol levels. This was most pronounced in the strains able to grow in the presence of 20% ethanol on plates. All of these strains showed high maximal ethanol accumulation and 50% produced a final ethanol level higher than 18% (v/v). On the other hand, the general correlation between the two traits showed only weak significance (Spearman one-tailed test: 90% confidence interval, P-value = 0.0984). This suggested that the genetic basis of the two traits was at least partially different.

### Isolation of a superior segregant of CBS1585

**Figure 2:**
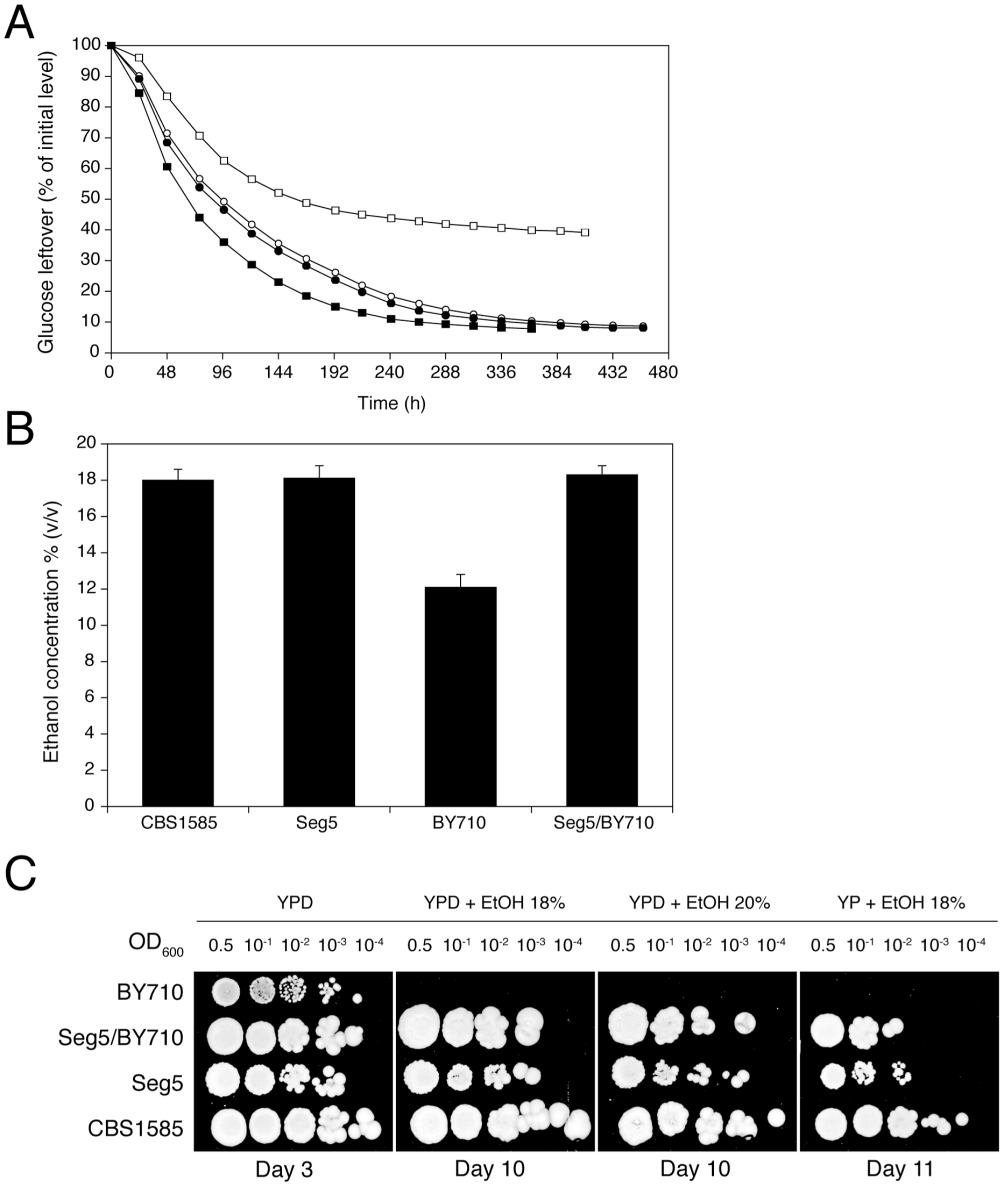
Maximal ethanol accumulation capacity and ethanol tolerance of cell proliferation in the superior parent and its segregant. (A) Identification of a segregant with the same high ethanol accumulation capacity of CBS1585. A segregant, Seg5 (n), derived from CBS1585 (2n) showed better attenuation of the fermentation medium compared to the laboratory strain BY710. The diploid (Seg5/BY710) showed similar final attenuation as the superior strains CBS1585 and Seg5. Strains: (•) Seg5, (○) CBS1585, (▪) Seg5/BY710 and (□) BY710. (B) Maximal ethanol production capacity in 250 mL of YP+33% glucose at 25°C. The strains CBS1585 (2n), Seg5 (n), Seg5/BY710 (2n) showed much higher ethanol accumulation capacity compared to BY710 (n). (C) Growth assays on plates containing YP or YPD plus ethanol (18 and 20% v/v). The strains CBS1585 (2n), Seg5 (n), Seg5/BY710 (2n) showed much higher ethanol tolerance of cell proliferation compared to BY710 (n).

The diploid sake strain CBS1585 was sporulated and stable mating type **a** and α segregants were obtained indicating heterothallism of the parent strain. Ten segregants were phenotyped in small-scale VHG semi-static fermentations. A segregant, Seg5 (MATa), was identified, which showed the same fermentation profile (Figure 2A) and maximal ethanol accumulation capacity as its parent strain, CBS1585 (Figure 2B). The laboratory strain BY710 (derived from BY4742; same genotype: *Matα his3Δ1 leu2Δ0 ura3Δ0 lys2Δ0*) showed a lower fermentation rate and also a much lower maximal ethanol accumulation capacity, which was only around 12% (v/v) (Figure 2A and 2B). The **a** mating type of the Seg5 strain was stable and FACS analysis confirmed that its DNA content was half that of its diploid parent CBS1585 (data not shown). We have crossed Seg5 with BY710 to obtain the diploid Seg5/BY710, which showed a similar high fermentation rate (Figure 2A) and high ethanol accumulation capacity (Figure 2B) as the original CBS1585 diploid strain. Growth assays on solid media, with or without glucose, and containing different levels of ethanol, showed that CBS1585, Seg5 and Seg5/BY710 had a similar ethanol tolerance of cell proliferation whereas the laboratory strain (BY710) was much more sensitive (Figure 2C). These results indicate that the two ethanol tolerance traits are dominant characteristics in the strain backgrounds used.

### Comparison between ethanol tolerance of cell proliferation on solid nutrient plates and maximal ethanol accumulation capacity in fermentation

**Figure 3:**
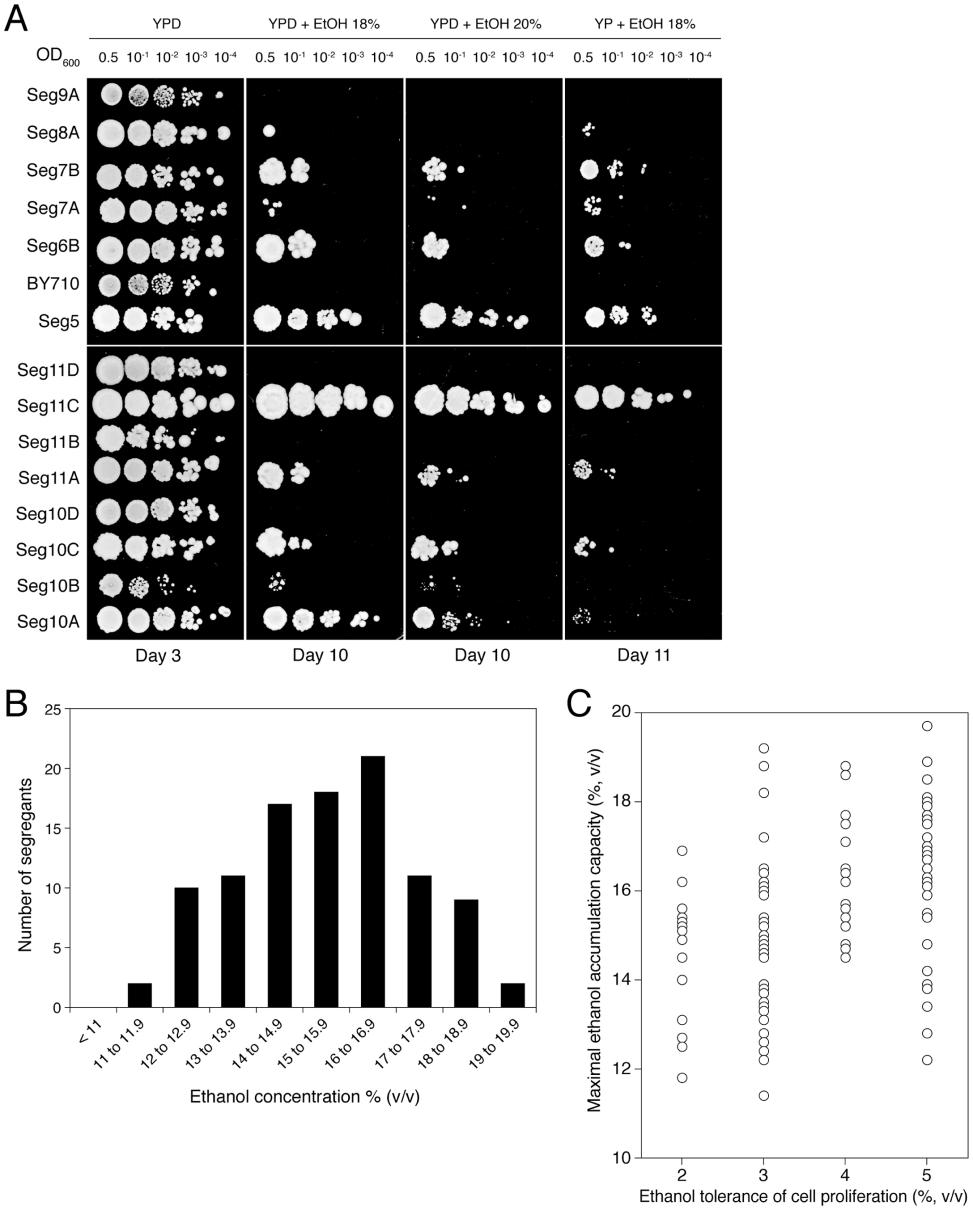
Maximal ethanol accumulation capacity and ethanol tolerance of cell proliferation in meiotic segregants. (A) Cell proliferation assays on solid media containing YP or YPD plus ethanol (18% and 20% v/v). Stationary phase cells were diluted ten-fold from OD600: 0.5 and 4 µL were spotted on the different media. Seg5 (n) showed much higher ethanol tolerance than BY710 (n) and the segregants derived from the diploid Seg5/BY710 presented different cell proliferation capacity (e.g. Seg11C showed high ethanol tolerance whereas Seg11D was ethanol sensitive). The performance of the segregants in this assay received scores from 0 till 5 according to the growth in the different dilutions. (B) Distribution of maximal ethanol accumulation capacity within 101 meiotic segregants derived from Seg5/BY710. The 101 segregants were preselected based on the assay for ethanol tolerance of cell proliferation (minimum score of 2). The semi-static fermentations were performed in 250 mL of YP+33% glucose at 25°C. (C) Ethanol tolerance of cell proliferation (X-axis) versus maximal ethanol accumulation capacity (Y-axis), expressed as maximal ethanol titer reached, in the 101 segregants. The score for ethanol tolerance of cell proliferation was determined as explained in (A).

We have investigated whether ethanol tolerance as determined by the classical assays of cell proliferation on solid nutrient plates containing different levels of ethanol, correlates with maximal ethanol accumulation capacity in fermenting cells in the absence of cell proliferation. For that purpose, Seg5 was crossed with BY710, the Seg5/BY710 diploid sporulated and the segregants were first plated on solid media containing glucose and/or ethanol (18% to 20% v/v). Figure 3A shows a representative result. The haploid parent Seg5 showed high tolerance of cell proliferation to ethanol whereas the laboratory strain BY710 was much more ethanol sensitive. Among the segregants we could observe some with very high ethanol tolerance (e.g. Seg 11C), some with intermediate tolerance (e.g. Seg 10A) and others that were as ethanol sensitive as the laboratory strain (e.g. Seg11D). Out of 301 segregants evaluated in this way, 101 segregants showed moderate to high ethanol tolerance, whereas about half of the segregants (48.8%) could not grow at all on plates containing 18 or 20% ethanol (v/v). In the first category, 32 segregants showed an ethanol tolerance level as high as Seg5. Hence, about 1 in 9 segregants showed the same high ethanol tolerance as the superior parent. If we suppose random segregation of the loci and no epistasis, this ratio predicts three independent loci as being involved in determining the high ethanol tolerance of Seg5 compared to the laboratory strain BY710.

Subsequently, we tested 15 ethanol sensitive segregants (similar to Seg11D of Figure 3A) by fermentation in 250 mL of YP+33% (w/v) glucose. All 15 segregants clearly showed poor fermentation performance, with a low ethanol accumulation capacity (<14% v/v) (not shown). This suggests that there is a correlation between ethanol tolerance as measured by the cell proliferation assays on solid nutrient plates and maximal ethanol accumulation capacity in VHG fermentation, at least for the ethanol sensitive strains. Hence, to reduce the high workload required for phenotyping all segregants in fermentations, we tested in the small-scale fermentations only the 101 segregants that showed moderate to high ethanol tolerance in the growth assays on solid nutrient plates. We are aware that the strains with poor ethanol tolerance of cell proliferation may contain mutant genes that compromise maximal ethanol accumulation capacity or that when these strains show relatively high maximal ethanol accumulation capacity, they may contain (in part) different mutant alleles than the strains with high ethanol tolerance of cell proliferation. The main purpose of this work, however, was to identify the first set of major causative genes determining maximal ethanol accumulation capacity and this is the main reason why we continued first with the strains preselected for medium to high ethanol tolerance of growth.

The distribution of maximal ethanol accumulation capacity among the 101 segregants, as tested in semi-static small-scale fermentations in 250 mL of YP+33% (w/v) glucose, is shown in Figure 3B. We have also compared ethanol tolerance of cell proliferation and maximal ethanol accumulation capacity for the 101 segregants. The results are shown in Figure 3C. They are similar to the results obtained for the 68 natural and industrial yeast strains (Figure 1B) in two aspects. First, irrespective of the ethanol tolerance of cell proliferation, the segregants show a wide range of ethanol accumulation capacities. This confirms that the correlation between the two properties is weak. Second, the segregants with a higher ethanol tolerance of cell proliferation show a tendency towards higher ethanol accumulation capacity. The latter effect is less pronounced than with the selection of strains in Figure 1B, but this can be due to the fact that the poorest segregants for ethanol tolerance of cell proliferation have already been eliminated for the high-gravity fermentation experiments.

Only 22 segregants produced ethanol titres higher than 17% (v/v), similar to the ethanol production of Seg5 and Seg5/BY710. If we assume that all ethanol sensitive segregants, as determined by growth assays on solid nutrient plates, also display poor maximal ethanol accumulation, we have a ratio of one superior strain in ±14 segregants (301/22 = 13.7). Assuming random segregation of the QTLs and no epistasis, this ratio is consistent with four independent loci being responsible for the superior ethanol accumulation capacity of Seg5 compared to the BY710 control strain. We constructed several diploids by crossing the four best performing segregants but none of those showed higher ethanol accumulation capacity than the original CBS1585 diploid strain (data not shown).

### QTL mapping by pooled-segregant whole-genome sequence analysis

We have performed genetic mapping of the two polygenic traits: on the one hand, high ethanol accumulation capacity in fermenting cells in the absence of cell proliferation, using the 22 best-performing segregants (pool 1) as determined in semi-static VHG fermentations, and on the other hand, tolerance of cell proliferation to high ethanol levels, using the 32 segregants (pool 2) that showed the best growth on solid nutrient media containing 18 to 20% (v/v) ethanol. The two pools had 12 segregants in common. Identification of the QTLs was performed by pooled-segregant whole genome sequence analysis [5], [6], [8], [9]. Genomic DNA was sent to two independent companies (GATC Biotech, Konstanz, and BGI, Hong Kong) for custom whole-genome sequence analysis with an average depth of ∼38 by the Illumina platform. Other sequencing parameters are summarized in the Methods section.

Sequence analysis of the genome of the superior parent Seg5 and comparison to S288c, allowed us to select 48,512 high-quality SNPs after filtering for sufficient coverage (≥20 times) and ratio (≥80%) [5], [22]. The coverage of at least 20 times was based on previous findings that a 20-fold sequencing coverage is sufficient to compensate for errors by the number of correct reads [23]. The ratio of at least 80% was chosen based on the plots of the SNPs between the two parent strains [5]. We also mapped the reads to the assembled sequence for the Kyokai n°7 strain available in the *Saccharomyces* genome database [24]. We were able to map about 20,000 additional reads to this sequence and 93% of the total read pairs aligned with proper distance and orientation to the Kyokai n°7 assembly, while only 87% of the read pairs mapped in the same way to S288c. We also identified the sake strain specific genes *AWA1* and *BIO6* [24], which further confirmed that CBS1585 belongs to the sake cluster of *S. cerevisiae* strains.

**Figure 4:**
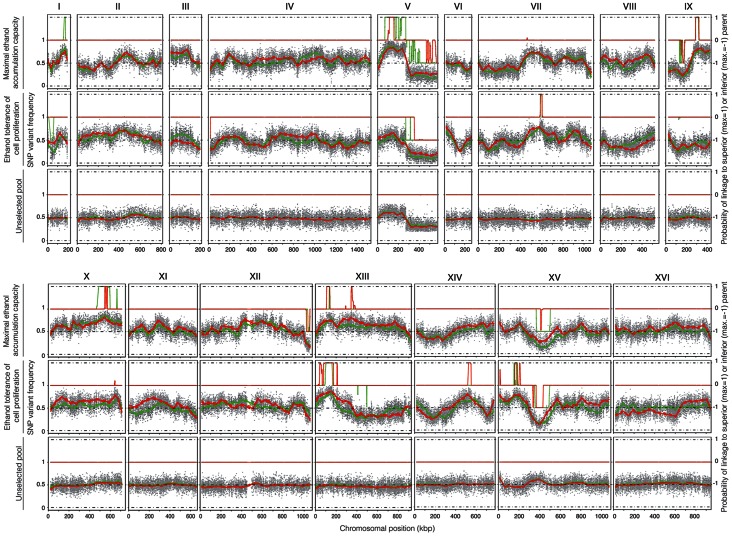
QTL mapping of maximal ethanol accumulation capacity (pool 1) and high ethanol tolerance of cell proliferation (pool 2). 22 selected segregants (pool 1) with high ethanol accumulation capacity and 32 selected segregants (pool 2) with high ethanol tolerance of cell proliferation were pooled for whole genome sequencing analysis, which was performed by two independent companies utilizing the Illumina platform (BGI in green and GATC Biotech in red). An unselected pool composed of 237 segregants (pool 3) was also sequenced twice to assess proper segregation of all chromosomes and possible linkage to inadvertently selected traits. The probability of linkage to the superior or the inferior parent, as determined with the HMM, is indicated on the right.

Genomic DNA was extracted from the two selected pools, containing 22 and 32 segregants, respectively, and also from an unselected pool, composed of 237 segregants (pool 3) in order to assess proper segregation of all chromosomes and possible links to inadvertently selected traits, such as sporulation capacity or spore viability. After sequence analysis, the SNP variant frequency was plotted against the chromosomal position (Figure 4). Upward deviations from the mean of 0.5 identify QTLs linked to the superior parent Seg5, while downward deviations identify QTLs linked to the inferior parent BY710. In most areas of the genome, and especially in the QTL areas, the independent sequence analysis by the two companies matched well, which confirms the robustness of the pooled-segregant whole-genome sequencing technology. Only in some selected areas the matching was poorer, which may be due to the low pool sizes. The SNP variant frequencies were smoothed using a Linear Mixed Model (LMM) framework [5] and the putative QTLs were identified by applying a Hidden Markov Model (HMM) similar to the one implemented in the FastPHASE package [25]. For each polymorphism, the HMM had three possible states: (i) a link with the superior parent (Seg5), (ii) a link with the inferior parent (BY710) and (iii) no link (background level). The SNP frequencies for each pool of segregants, analysed with the HMM, were assigned probability scores, that indicated to which state (Seg5, BY710 or background) they belonged and hence identified the QTLs, linked to either the superior parent (Seg5) or to the inferior parent (BY710).

The smoothed data of the SNP variant frequency and the Probability of linkage values obtained by HMM analysis with the selected pools 1 and 2 and the unselected pool 3, are shown in Figure 4. The QTLs identified with the HMM approach are listed in Tables 2 and 3 for pools 1 and 2, respectively. SNPs were considered significantly linked to the superior or inferior parent strain when the Probability of linkage was higher than 0.95 or lower than −0.95, respectively. The QTLs were numbered according to their position in the genome starting from chromosome I, independently of the trait (Tables 2 and 3).

**Table 2:** QTLs identified for maximal ethanol accumulation capacity (pool 1, 22 segregants) by pooled-segregant whole-genome sequencing.

QTL	Chr.	Genomic position (bp)	Nr. SNPs with significant linkage	Average Probability of linkage	Association with parent	Presence in pool 2
2	I	168455–179051	30	0.996868	Seg5	No
3	V	69939–166080	348	0.999346	Seg5	No
4	V	178671–198538	84	0.999191	Seg5	No
5	V	230340–269314	187	0.997819	Seg5	No
7	X	136210–175751	148	−0.986817	BY	No
8	X	288210–321763	107	0.999024	Seg5	No
9	X	486491–594119	230	0.99672	Seg5	No
10	XII	1022570–1053429	94	−0.999094	BY	Weak
12.1	XIII	109860–137864	47	0.994056	Seg5	Yes
13	XIII	346583–352695	27	0.991967	Seg5	Weak
17.1	XV	372007–494421	247	−0.999883	BY	Yes

Eight QTLs were associated with the genome of the superior parent Seg5 and three QTLs linked to the genome of the inferior parent BY710. The chromosomal position of each QTL, the number of SNPs with significant linkage and the average Probability of linkage of all significant SNPs in the QTL are indicated. All QTLs indicated had a significant Probability of linkage >0.95 when linked to the Seg5 parent or <−0.95 when linked to the BY parent. QTLs 1, 6, 11, 14, 15 and 16 were found only in pool 2 (see Table 3) whereas QTLs 12 and 17 were common for both pools and designated 12.1 and 12.2 or 17.1 and 17.2 depending on the pool.

**Table 3:** QTLs identified for tolerance of cell proliferation to high ethanol (pool 2, 32 segregants) by pooled-segregant whole-genome sequencing.

QTL	Chr.	Genomic position (bp)	Nr. SNPs with significant linkage	Average Probability of linkage	Association with parent	Presence in pool 1
1	I	29970–55793	83	-0.998124	BY	Weak
6	VII	585062–600706	50	0.99851	Seg5	Weak
11	XIII	43152–51596	37	0.97562	Seg5	Weak
12.2	XIII	79761–173678	183	0.998144	Seg5	Yes
14	XIV	525370–549448	70	0.997764	Seg5	No
15	XV	161704–184072	59	0.997942	Seg5	Weak
16	XV	205844–210327	26	0.970977	Seg5	Weak
17.2	XV	356119–487809	285	-0.99949	BY	Yes

There are six QTLs linked to the genome of the superior parent Seg5 and two QTLs linked to the genome of the inferior parent BY710. The chromosomal position of each QTL, the number of SNPs with significant linkage and the average Probability of linkage of all significant SNPs in the QTL are indicated. All QTLs indicated had a significant Probability of linkage >0.95 when linked to the Seg5 parent or <−0.95 when linked to the BY parent. QTLs 2, 3, 4, 5, 7, 8, 9, 10 and 13 were found only in pool 1 (see Table 2) whereas QTLs 12 and 17 were common for both pools and designated 12.1 and 12.2 or 17.1 and 17.2 depending on the pool.

The unselected pool 3 (237 segregants) showed ±50% SNP variant frequency in most of the genome and thus no evidence of any QTLs (Figure 4). The only exception was the right arm of chromosome V which was preferentially inherited from the BY parent strain. Comparison with the data of the selected pools, suggested some weak linkage with the genome of the BY parent strain in this part of chromosome V. Because of the weak linkage this was not retained for further analysis. Crosses of Seg5 with other BY strains did not show aberrant segregation of the right arm of chromosome V (results not shown). The results obtained with the unselected pool show that the QTLs identified for the two ethanol tolerance traits were not due to linkage with inadvertently selected traits, such as sporulation capacity or spore viability.

The QTLs identified with the selected pools 1 and 2 showed two common QTLs (on chr XIII and chr XV). They were called 12.1 and 17.1 for pool 1 and 12.2 and 17.2 for pool 2. It has to be emphasized that the ‘common’ character of these QTLs is only based on their common location in the genome. In principle, they could be located in the same place on a chromosome but caused by a different causative gene. Moreover, the QTLs 15 and 16 (pool 2) were also present in pool 1 as minor putative QTL of which the significance could not be demonstrated with the current number of segregants (Probability of linkage <0.95). Other minor putative QTLs of which the significance could not be demonstrated with the current number of segregants (Probability of linkage <0.95) were present in pool 1 and pool 2. They were also seen with the smoothed data and the HMM analysis (Figure 4) (e.g. on chromosome VII). There was no indication for linkage of the areas with the sake strain specific genes *AWA1* and *BIO6* to one or both of the ethanol tolerance traits.

### Identification of causative genes in QTLs of pool 1

**Figure 5:**
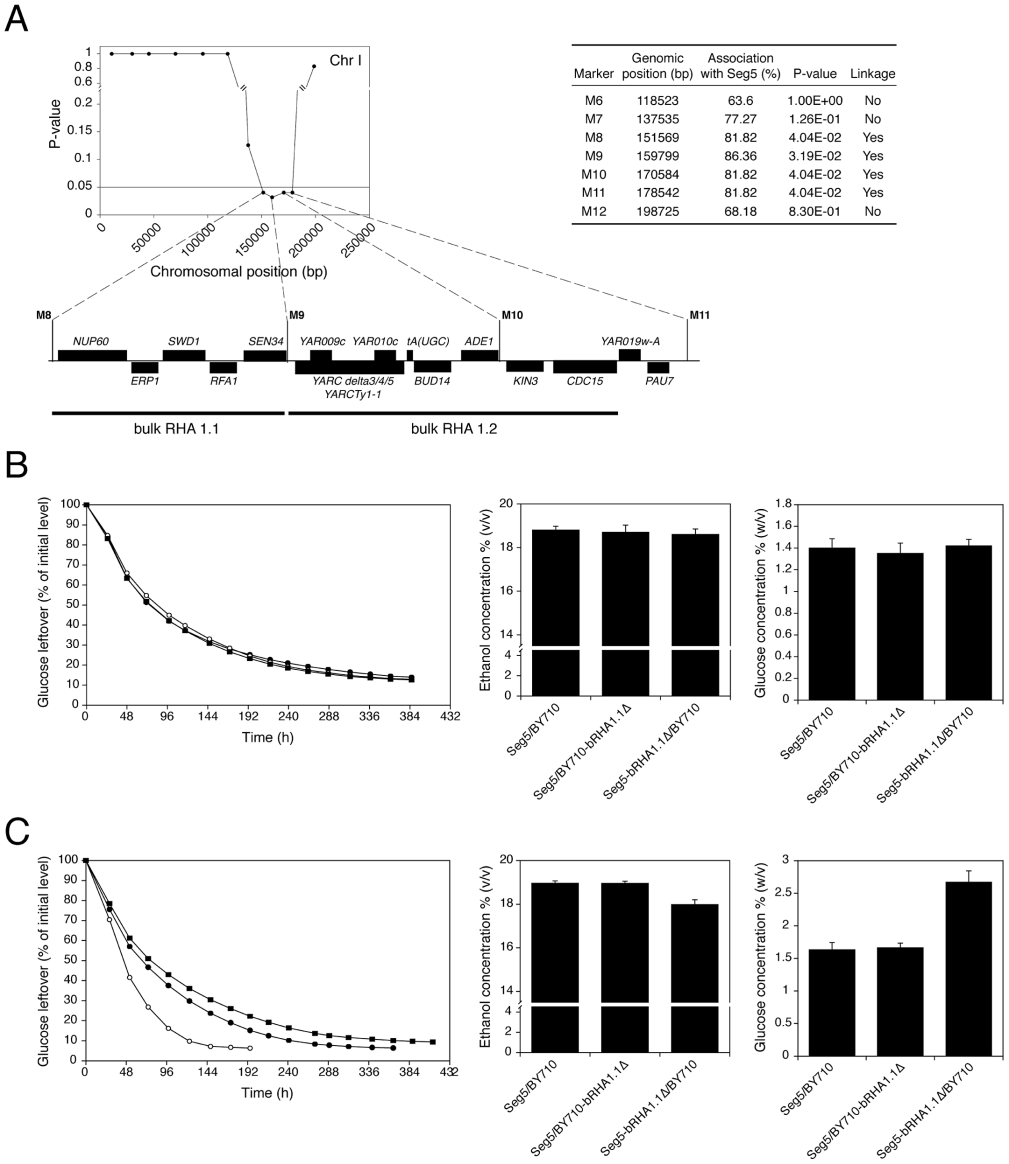
Fine-mapping and bulk RHA of QTL2. (A) Genes present in QTL2 (pool 1), located on chromosome I, as determined by markers scored in the 22 segregants individually. (B) Bulk RHA (bRHA 1.1) of genes *NUP60*, *ERP1*, *SWD1*, *RFA1* and *SEN34*. Two heterozygous diploids for the five genes were constructed: Seg5/BY710-bRHA1.1Δ (○) and Seg5-bRHA1.1Δ/BY710 (▪). These two diploids were compared with the original strain Seg5/BY710 (•) in semi-static fermentations performed in 250 mL of YP+33% glucose at 25°C. (C) Bulk RHA (bRHA 1.2) of genes *YARCdelta3/4/5*, *YARCTy1-1*, *YAR009c*, *YAR010c*, *tA(UGC)*, *BUD14*, *ADE1*, *KIN3*, and *CDC15*. Two heterozygous diploids for the previous genes were constructed: Seg5/BY710-bRHA1.2Δ (○) and Seg5-bRHA1.2Δ/BY710 (▪). These two diploids were compared with the original strain Seg5/BY710 (•) in semi-static fermentations performed in 250 mL of YP+33% glucose at 25°C.

We have analysed in detail two QTLs (2 and 3) involved in high ethanol accumulation capacity (pool 1) because this trait is more relevant in industrial fermentations and because these two QTLs were among those with the strongest linkage. QTL2 is located on chromosome I and was fine-mapped by scoring selected markers in the 22 individual segregants. This reduced the length of the QTL to the area between chromosomal positions 151 kb and 178 kb (P-value<0.05) (Figure 5A). The association percentage of the markers, their genomic positions, the respective P-values and the genes located in the putative QTL 1 are shown in Figure 5A.

Nearly all genes present in the centre of the QTL had at least on polymorphism either in the ORF, promotor or terminator. Hence, it was not possible to exclude on this basis a significant number of genes as candidate causative genes. Because of the large number of candidate genes and the high workload of the phenotyping for maximal ethanol accumulation capacity, we have introduced a modification of the Reciprocal Hemizygosity Analysis (RHA) methodology, which has been used previously for identification of causative genes [10]. Instead of testing one candidate gene at a time, we first evaluated a series of adjacent genes by ‘bulk RHA’. For that purpose a set of adjacent genes was deleted directly in the heterozygous diploid background (Seg5/BY710) so as to obtain the two reciprocally deleted hemizygous diploids of which the phenotype was subsequently compared. The first block of genes (bRHA 1.1) deleted, consisted of *NUP60*, *ERP1*, *SWD1*, *RFA1* and *SEN34*. The two reciprocally deleted diploid strains were tested by fermentation in YP+33% (w/v) glucose, to address the effect of the Seg5 and BY710 alleles on ethanol accumulation capacity. The results showed no difference in the fermentation profile and maximal ethanol accumulation (Figure 5B), suggesting that none of these five genes were causative genes. There was also no difference in fermentation profile and maximal ethanol accumulation with the hybrid parent strain Seg5/BY710, further supporting that these genes did not influence these phenotypes.

The second block of genes tested consisted of *YARCdelta3/4/5*, *YARCTy1-1*, *YAR009c*, *YAR010c*, *tA(UGC)A*, *BUD14*, *ADE1*, *KIN3* and *CDC15* (bRHA 1.2) (Figure 5A). In this case there was a clear reduction of the fermentation rate and maximal ethanol accumulation when the alleles of the Seg5 strain were absent compared to absence of the BY710 alleles (Figure 5C). Glucose leftover correlated inversely with final ethanol titer. This suggested the presence of one or more causative genes in this region. Moreover, the fermentation rate was higher in the hemizygous strain where the BY710 alleles were absent compared to the hybrid parent strain Seg5/BY710, indicating that one or more of the BY710 alleles had a negative effect on this phenotype.

**Figure 6:**
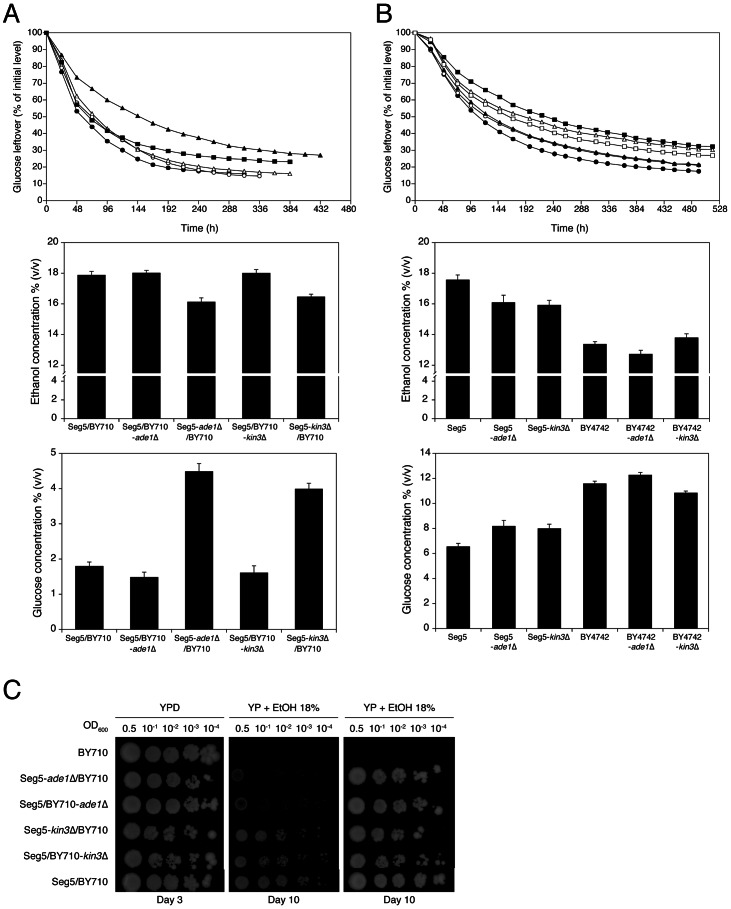
Single gene RHA and loss of function assessment for the causative genes *ADE1* and *KIN3* in QTL2. (A) RHA of genes *ADE1* and *KIN3*. The diploid strain Seg5/BY710 (•) had *ADE1* or *KIN3* deleted in one of the alleles separately. The resulting strains Seg5/BY710-ade1Δ (○), Seg5-ade1Δ/BY710 (▴), Seg5/BY710-kin3Δ (Δ) and Seg5-kin3Δ/BY710 (▪) were compared with the original diploid Seg5/BY710 (•) in semi-static small-scale fermentations in YP+33% glucose at 25°C. The deletion of the alleles present in Seg5 resulted in diploids with lower ethanol accumulation capacity in comparison to the original strain and the deletion of the alleles from BY710. (B) *ADE1* and *KIN3* loss-of-function assays. The genes *ADE1* and *KIN3* were deleted in the haploid strains Seg5 (•) and BY4742 (Δ) separately. The strains Seg5-ade1Δ (○), Seg5-kin3Δ (▴), BY4742-ade1Δ (▪) and BY4742-kin3Δ (□) were evaluated by semi-static fermentations in 250 mL of YP+33% glucose at 25°C. (C) Determination of ethanol tolerance of cell proliferation with the hybrid diploid strains Seg5/BY710-ade1Δ, Seg5-ade1Δ/BY710, Seg5/BY710-kin3Δ and Seg5-kin3Δ/BY710.

*YARCdelta3/4/5, YARCTy1-1, YAR009c and YAR010c* are transposable elements, while tA(UGC)A encodes one of the sixteen tRNAs for the amino acid alanine. *BUD14* is involved in bud-site selection [26], *ADE1* is involved in *de novo* purine biosynthesis [27], *KIN3* encodes a non-essential serine/threonine protein kinase involved in a.o. DNA damage repair [28] and *CDC15* encodes a protein kinase involved in control of the cell division cycle [29]. In order to identify the genes(s) involved in ethanol accumulation capacity, we investigated the most likely candidate genes individually with the classical one-gene RHA [10]. Involvement of the transposable elements appeared unlikely and was not evaluated by RHA. The other genes, *BUD14*, *ADE1*, *KIN3* and *CDC15*, have polymorphisms (SNPs and/or indels) within their ORFs and/or promoter regions. RHA with the genes *ADE1* and *KIN3* showed that deletion of the Seg5 alleles resulted in strains with clearly lower ethanol accumulation capacity and higher glucose leftover compared to the strain with deletion of the respective BY allele, indicating that *ADE1* and *KIN3* are causative genes for high ethanol accumulation capacity in Seg5 (Figure 6A). For both genes, the hybrid parent strain Seg5/BY710 behaved in a similar way as the strain with the deleted BY710 allele. For *CDC15* and *BUD14* there was no difference in the performance of the two reciprocally deleted diploid strains (not shown). Deletion of *ADE1* and *KIN3* in the Seg5 and BY backgrounds caused a more pronounced effect in the Seg5 sake genetic background (Figure 6B).

The causative genes *ADE1* and *KIN3* were located in QTL2, which was not linked with ethanol tolerance of cell proliferation. When we tested the hybrid diploid strains previously used in RHA for maximal ethanol accumulation for determination of ethanol tolerance of cell proliferation, we could indeed not observe any significant difference between the two strains (Figure 6C). This confirms that these causative genes are specific for maximal ethanol accumulation capacity and that the genetic basis of the two ethanol tolerance traits is indeed partially different.

**Figure 7:**
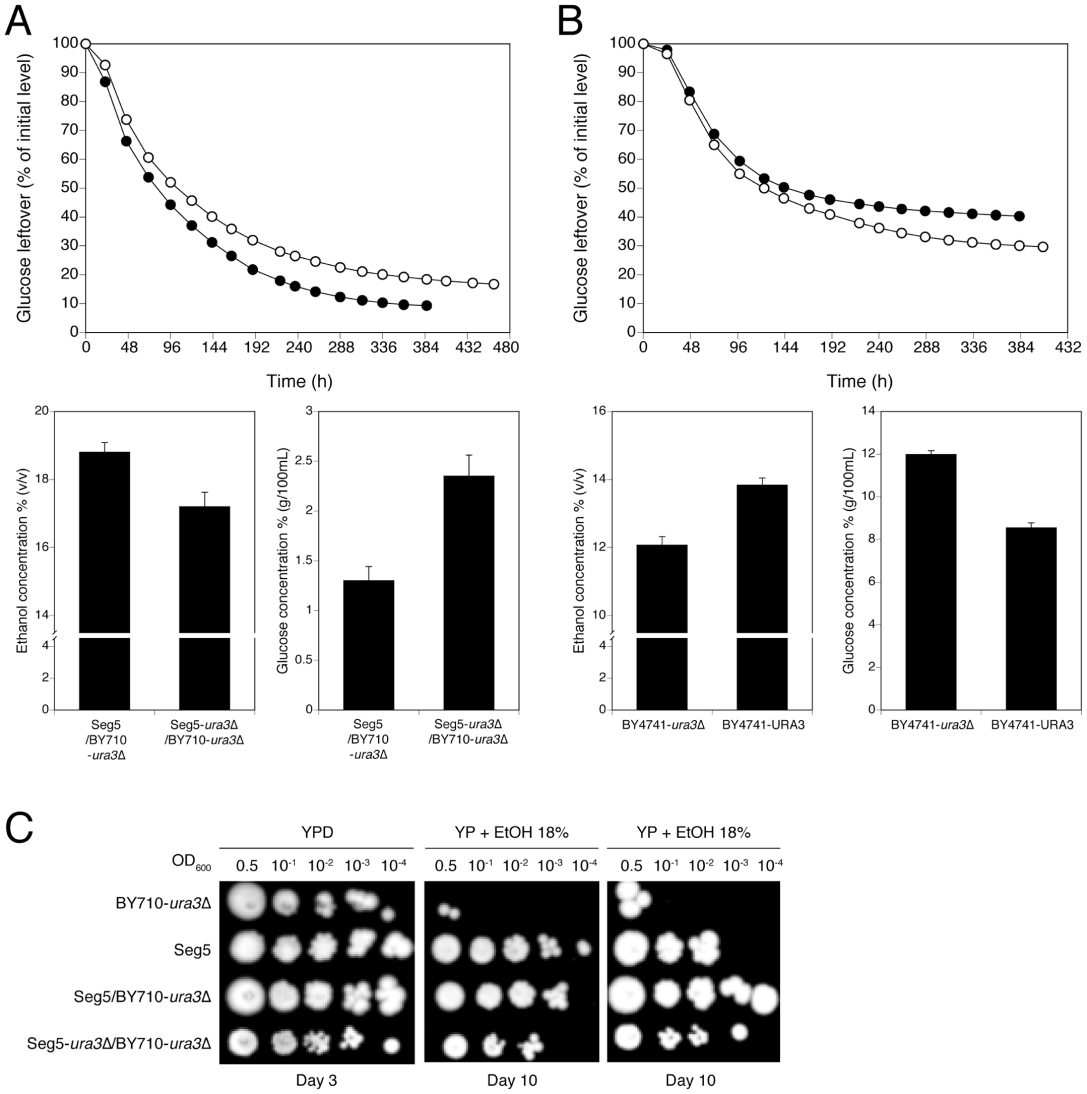
Loss of function assessment and complementation assay with the causative gene *URA3* in QTL3. (A) *URA3* loss-of-function assay. The strain Seg5/BY710 (•) had its *URA3* copy deleted, Seg5-ura3Δ/BY710 (○). Both strains were tested in 250 mL of YP+33% glucose at 25°C. (B) *URA3* complementation study. The *URA3* auxotrophic strain BY4741-ura3Δ (▪) had the *URA3* gene inserted in its original position, BY4741-*URA3* (□). The performance of both strains were assessed by semi-static fermentations in 250 mL of YP+33% glucose at 25°C. (C) Determination of ethanol tolerance of cell proliferation with the hybrid diploid strains Seg5/BY710-ura3Δ, Seg5-ura3Δ/BY710-ura3Δ.

We also analysed in more detail QTL3, located on chromosome V. In the same chromosomal region, Swinnen et al. [5] previously identified *URA3* as a causative gene in tolerance of cell proliferation to high ethanol levels of VR1, a Brazilian bioethanol production strain, in comparison with BY4741 as inferior parent strain. Since we crossed Seg5 with an *ura3* auxotrophic laboratory strain (BY710), we first tested whether deletion of *URA3* in Seg5 affected maximal ethanol accumulation in this genetic background. The fermentation profile and maximal ethanol accumulation of the strain Seg5-ura3Δ/BY710-ura3Δ (which is thus homozygous for *ura3Δ*) compared with the Seg5/BY710-ura3Δ diploid (which is heterozygous for *ura3Δ*) are shown in Figure 7A. Double deletion of *URA3* resulted in a strain with a reduced ethanol fermentation rate, lower maximal ethanol accumulation and higher glucose leftover. We have also tested the effect of introducing *URA3* in the *ura3* auxotrophic strain BY4741, which accumulates only low amounts of ethanol under VHG conditions (±12% v/v). Introduction of *URA3* enhanced the fermentation rate in the later stages of the fermentation and resulted in a clearly higher maximal ethanol titer and lower glucose leftover (Figure 7B). These results show that *URA3* positively affects maximal ethanol accumulation capacity. The *URA3* gene was located in QTL3, which was not significantly linked with ethanol tolerance of cell proliferation. When we tested the hybrid diploid strains previously used in RHA for maximal ethanol accumulation for determination of ethanol tolerance of cell proliferation, we observed slightly better growth for the strain with the *URA3* allele from Seg5 (Figure 7C). This confirms that *URA3* has only a minor contribution to this phenotype in this genetic background and suggests that the very weak upward deviation in the SNP variant frequency plot observed in this position for ethanol tolerance of cell proliferation might have been due to the *URA3* gene.

### Occurrence of the SNPs in the causative genes *ADE1* and *KIN3* in other yeast strains

Comparison of the sequence of *ADE1* and *KIN3* in Seg5 and BY710 (S288c background) revealed a C to T transition in the promoter of *ADE1* and a C to T transition in the promoter of *KIN3* as well as three synonymous transition mutations in the ORF of *KIN3*. We have checked the presence of these SNPs in the *ADE1* and *KIN3* genes of 36 yeast strains of which the whole genome sequence has been published. The results are shown in Table 4. (Among the 36 strains there were additional SNPs compared to S288c, which were not present in Seg5. These SNPs are not shown). The C to T change at position 169227 in *ADE1* is present only in two other strains, Kyokai nr. 7 and UC5. Both strains are sake strains and these strains are known to have superior maximal ethanol accumulation capacity. Sake fermentation produces the highest ethanol level of all yeast fermentations for production of alcoholic beverages [21]. The SNPs in *KIN3* of Seg5 at positions 170564 and 170945 are present in many other strains. Interestingly, however, the two other SNPs in *KIN3* of Seg5, at positions 170852 (in the ORF) and 171947 (in the promoter) are not present in *KIN3* of any one of the 36 sequenced strains and therefore may be rather unique.

**Table 4:** Occurrence of the SNPs in the causative genes *ADE1* and *KIN3* in other yeast strains.

		SNP					
		*ADE1*	*KIN3*				
		Prom.	ORF				Prom.
		169227	170564		170852	170945	171947
BY710 (∼BY4742)	This study	C	G		C	A	C
Seg5 (sake)	This study	**T**	**A**		**T**	**G**	**T**
Kyokai no.7 (sake)	BABQ01000003	**T**	G		C	A	C
EC9-8	AGSJ01000959	C	G		C	A	C
Lalvin_QA23	ADVV01000003	C	**A**		C	A	C
VIN13	ADXC01000003	C	**A**		C	A	C
JAY291	ACFL01000304	C	**A**		C	A	C
L1528	Liti et al. 2009	C	**A**		C	A	C
ForstersB*	AEHH01000001	C	G	**A**	C	A	C
Forsters0	AEEZ01000002	C	G		C	A	C
AWRI 1631	ABSV01000027	C	**A**		C	A	C
AWRI 796	ADVS01000002	C	**A**		C	A	C
UC5 (sake)	AFDD01000983	**T**	G		C	A	C
YPS128	Liti et al. 2009	C	**A**		C	A	C
T7	AFDE01000131	C	**A**		C	**G**	C
YJSH1	AGAW01000003	C	G		C	**G**	C
ZTW1	AMDD01000002	C	G		C	A	C
Y12	Liti et al. 2009	C	G		C	**G**	C
VL3	AEJS01000003	C	**A**		C	A	C
CBS 7960	AEWL01000708	C	**A**		C	A	C
T73	AFDF01002558	C	**A**		C	A	C
DBVPF1106	Liti et al. 2009	C	**A**		C	A	C
PW5	AFDC01000005	C	G		C	**G**	C
Sigma1278b	ACVY01000029	C	G		C	**G**	C
RM11-1a	AAEG01000015	C	**A**		C	A	C
CEN.PK113-7D	AEHG01000254	C	G		C	A	C
Y55	Liti et al. 2009	C	G		C	**G**	C
W303	ALAV01000008	C	G		C	A	C
SK1	Liti et al. 2009	C	G		C	**G**	C
UWOPS83-787_3	Liti et al. 2009	C	**A**		C	**G**	C
UWOPS03-461.4	Liti et al. 2009	C	**A**		C	A	C
UWOPS87-2421	Liti et al. 2009	C	G		C	**G**	C
DBVPG1373	Liti et al. 2009	C	**A**		C	A	C
DBVPG6044	Liti et al. 2009	C	G		C	**G**	C
DBVPG6765	Liti et al. 2009	C	**A**		C	A	C
YJM789	AAFW02000160	C	**A**		C	A	C
YJM975	Liti et al. 2009	C	**A**		C	A	C
YJM269	AEWN01000622	C	**A**		C	A	C
						
BY710 variant		34	15		36	26	36
Seg5 variant		**2**	**20**		**0**	**10**	**0**

* The strain ForstersB is heterozygous and has both variants.

The SNPs present in Seg5 compared to S288c were checked in 36 strains of which the whole genome sequence has been published. (SNPs present in the other strains compared to S288c, but not in Seg5, are not indicated).

## Discussion

Tolerance to high ethanol levels is an exquisite characteristic of the yeast *Saccharomyces cerevisiae* and no other microorganism has ever been reported to show higher ethanol tolerance. This unique property of yeast lies at the basis of the production of most alcoholic beverages and of ethanol as biofuel. In most studies, ethanol tolerance has been assayed by measuring cell proliferation in the presence of increasing ethanol levels. Although this assay is convenient for routine measurement and large-scale screenings, its true relevance for ethanol tolerance in yeast fermentation is unclear. Industrial yeast fermentations always start with an excess of fermentable sugar compared to other essential nutrients. As a result, the ethanol production rate in the second phase of the fermentation, the extent of attenuation of the residual sugar and the final ethanol titer reached are always achieved by stationary phase cells. In this work we have compared for the first time the genetic basis of maximal ethanol accumulation capacity in fermenting cells in the absence of cell proliferation with that of ethanol tolerance of cell proliferation. To avoid interference by the genetic background of the strain, we have used the same pool of segregants derived from one hybrid parent. The results of the QTL mapping by pooled-segregant whole-genome sequence analysis reveal a partial overlap between the genetic basis of the two traits. Although only two significant QTLs, 12.1/12.2 on Chr. XIII and 17.1/17.2 on Chr. XV appear identical, there were minor QTLs in pool 1 of which the significance could not be demonstrated with the current number of segregants (e.g. on Chr. VII and XV), which are likely overlapping with significant QTLs in the same position in pool 2. However, because of the lower number of segregants in pool 1, the P-value of these QTLs is not low enough for significance. It is also important in this respect to recall that the two pools had 12 segregants in common. A stronger argument for partial overlap between the genetic basis of the two traits could be made if two pools would be assembled not only with different segregants but containing in each pool only segregants that would not fit phenotypically in the other pool. This would have required, however, a large amount of additional experimental work.

Our work has shown that successful QTL mapping using pooled-segregant whole-genome sequence analysis can be performed with relatively low numbers of segregants. This is particularly important for elucidation of the genetic basis of complex traits of industrial importance, like maximal ethanol accumulation capacity, which require laborious experimental protocols for scoring. It has also shown that resorting to seemingly similar traits, like ethanol tolerance of cell proliferation, which can be scored easily with simple growth tests on plates, is not a valid alternative. On the other hand, there were several minor QTLs detected for the trait of maximal ethanol accumulation capacity, for which the significance could not be demonstrated with the number of segregants used. The ability to detect QTLs depends on the importance of the causative allele for establishing the trait and on the number of QTLs/causative alleles involved. Higher numbers of segregants will therefore always be useful to map minor QTLs and identify their causative alleles.

Detailed analysis of QTL 2 on Chr. I and QTL 3 on Chr. V identified three genes specifically linked to maximal ethanol accumulation capacity, which indicates that ethanol tolerance as relevant for maximal ethanol accumulation in fermentations cannot be fully assessed in a reliable way by simple growth tests on solid nutrient plates in the presence of ethanol. The identification of *KIN3* as a causative gene is striking because it reveals for the first time a role for DNA damage repair in ethanol tolerance as required for maximal ethanol accumulation. Moreover, the superior *KIN3* allele of Seg5 contained two SNPs, which were absent in the *KIN3* gene of all yeast strains of which the genome has been fully sequenced up to now, suggesting that they may be important for the exceptional ethanol accumulation capacity of the Seg5 strain and its diploid parent CBS1585. *KIN3* encodes a serine-threonine protein kinase, required for arrest at the G2/M-phase checkpoint in response to the DNA damage inducing agents MMS, cisplatin, doxorubicin and nitrogen mustard [28]. Involvement of Kin3 in the DNA damage response may be consistent with its requirement for tolerance to high ethanol levels. Ethanol was reported to be mutagenic and to induce single-strand DNA breaks in repair-deficient but not in repair-proficient yeast cells [30]. It was also reported to trigger chromatin condensation, fragmentation, and DNA cleavage in yeast, features suggestive of induction of apoptosis [31]. Mitochondrial DNA loss in yeast is induced by ethanol and mitochondrial DNA from more ethanol tolerant flor yeasts enhanced ethanol tolerance when transferred into a laboratory strain [32]. Also in mammalian cells, ethanol was shown to induce DNA damage and is a known carcinogen [33]. A role for DNA repair in protecting mammalian cells from ethanol-induced damage has been proposed [34]. It will be interesting to investigate to what extent maximal ethanol accumulation in yeast can be enhanced by further strengthening DNA damage repair capacity.

The case of *URA3* is remarkable. It encodes one of the most active enzymes, oritidine 5-phosphate decarboxylase (OCDase), that catalyzes the decarboxylation of oritidine 5-phosphate (OMP) to uridylic acid (UMP) [35], [36]. This is the sixth enzymatic step in the *de novo* biosynthesis of pyrimidines. Yeast strains lacking *URA3* need supplementation with uracil in the medium. Our previous work identified *ura3* [5] and several other auxotrophic mutations (unpublished results) as causative mutations for ethanol tolerance of cell proliferation in a cross of a Brazilian bioethanol production strain VR1 and the BY laboratory strain. We have now identified *ura3* as causative gene for maximal ethanol accumulation capacity in the cross of the sake strain CBS1585 and BY. However, in this genetic background *ura3* was not significantly linked to ethanol tolerance of cell proliferation. This indicates that the genetic basis of the latter property is dependent on the genetic background of the strain. A stronger capacity to generate the electrochemical potential required for symport, may for instance offset the ethanol sensitivity of the uptake of auxotrophic supplements.

Lower expression of auxotrophic genes, like *URA3*, or lower activity of the gene product, forces the yeast cells to take up most uracil using the uracil permease, Fur4, which is an active proton symporter [37]. Stress conditions, including nutrient starvation, can trigger degradation of Fur4 [38]. Hence, the requirement of *URA3* for maximal ethanol production capacity might be linked to nutrient starvation towards the end of the semi-anaerobic, high-gravity fermentation process, which can take up to 21 days. Uracil is likely depleted and/or its transporter Fur4 may be degraded because of the nutrient starvation conditions at the end of the fermentation. In addition, ethanol toxicity may also compromise the proton gradient, which is required for uptake by symport of uracil and protons from the medium. This type of inhibition was reported for amino acid uptake by the proton symporter Gap1 [39]. The reduction of maximal ethanol accumulation in *ura3* auxotrophic strains suggests that in general the active uptake of nutrients may be compromised by the increasing ethanol level at the end of the fermentation. Yeast cells have only one permease to transport uracil, Fur4, which may make this system more sensitive to ethanol inhibition compared to for instance amino acid transport, for which many transporters exist.

Another relevant factor may be the general fitness problem of *URA3* deleted strains. *URA3* auxotrophic strains (BY710-ura3Δ, BY4741-ura3Δ and Seg5-ura3Δ/BY710-ura3Δ) showed much less biomass production in the pre-cultures performed in YPD, YP+5% glucose, YP+10% glucose and during the fermentations in YP+33–35% glucose (OD600 around 12.4±2.68) whereas Seg5/BY710-ura3Δ (prototrophic) for example, had much higher cell densities (32.6±3.42 in stirred fermentations). Low cell densities contribute to a slow fermentation phenotype that is also associated with lower final ethanol levels. The importance of uracil supplementation and fitness problems related to uracil auxotrophic strains have been reported recently by Basso *et al*. [19].

We identified the *ADE1* allele in Seg5 by RHA as a superior allele for maximal ethanol accumulation capacity in high-gravity fermentation. As in the case of *URA3*, there was no link between *ADE1* and tolerance of cell proliferation to high ethanol levels. *ADE1* encodes a N-succinyl-5-aminoimidazole-4-carboxamide ribotide (SAICAR) synthetase, that is required for *de novo* purine biosynthesis [27]. *ADE* genes have not been connected previously to ethanol tolerance, but they have been linked to high sugar tolerance. In a genome-wide screen with the deletion strain collection, Ando *et al*. [40] identified three adenine biosynthetic genes (*ADE5*,*7*, *ADE6* and *ADE8*) as being required for tolerance to 30% (w/v) sucrose. These genes were not required for tolerance to high sorbitol and NaCl, indicating a specific role in high sugar tolerance. The *ADE* genes are involved in biosynthesis of purine and derived metabolites, such as ATP. Measurements of the ATP level revealed a reduction with two-fold in the *ade* mutants, indicating that inability to synthesize sufficient ATP could be related to the high sucrose stress sensitivity. Alternatively, in the *ade* mutants the STRE-controlled stress response gene, *HSP12*, which encodes a plasma membrane chaperone protein, was not induced under high-sucrose stress, as opposed to sorbitol and salt stress [40]. This suggests a possible defect in induction of stress protection factors as cause for the high-sucrose sensitivity and once more a specific role of *ADE* genes in high sugar stress. Osmotic stress is known to trigger the HOG-pathway [41]. Phosphorylation of Hog1, the central component of the HOG pathway, however, was normal under all three osmotic stress conditions in all *ade* mutant strains, suggesting that deficiency of the HOG pathway, or at least the osmosensing systems, was not involved in the sensitivity of the *ade* mutants [40]. Because we measured maximal ethanol accumulation in fermentations with a very high sugar level (33%, w/v, glucose), the link with the superior allele of *ADE1* in QTL2 (chr I) may be due to its importance for tolerance to high sugar stress. If this would be the reason why the superior *ADE1* allele of Seg5 supports higher ethanol accumulation under VHG conditions, it would explain why the *ADE1* gene was not linked to ethanol tolerance of cell proliferation as measured with pool 2, since the solid nutrient plates contain a low sugar level and a high ethanol level. The *ADE1* gene from the superior parent Seg5 did not have any mutation in the ORF compared to the sequence in the laboratory strain BY. However, one SNP was located in the promoter region of the Seg5 allele (Chr I: 169.228 bp - C/T). The promoter of *ADE1* is known to bear a hexanucleotide (5′ TGACTC 3′) element that is under amino acid control [27]. Although the mutation is not within that regulatory element, it is possible that it is affecting *ADE1* expression and thereby also high sugar tolerance.

In conclusion, our work has shown that successful QTL mapping with pooled-segregant whole-genome sequence analysis can be performed for traits of industrial importance, which require elaborate experiments to score the phenotype, using a relatively low number of segregants. We have identified for the first time genes required for maximal ethanol accumulation capacity in the absence of cell proliferation in fermenting yeast cells and have shown that the genetic basis of this trait is partially different from that of tolerance of cell proliferation to high ethanol levels. The superior alleles identified can be used for improvement of maximal ethanol accumulation capacity in industrial yeast strains for bioethanol production and for the production of alcoholic beverages. This improves attenuation of the sugar at the end of the fermentation, which enhances yield in industrial bioethanol production and reduces residual sugar levels in alcoholic beverages. A higher final ethanol level in bioethanol production reduces distillation costs and lowers the liquid volumes in the plant, which in turn reduces costs associated with cooling, heating, pumping and transport of liquid residue.

## Materials and Methods

### Strains and growth conditions

The *S. cerevisiae* strains utilized in this study are listed in Table S2. Yeast cells were grown with orbital agitation (200 rpm) at 30°C in YPD medium containing 1% (w/v) yeast extract, 2% (w/v) Bacto peptone and 2% (w/v) glucose.

### Small-scale VHG fermentations for determination of maximal ethanol accumulation capacity

VHG fermentations were performed in which the glucose concentration was raised to such an extent (33% w/v) that a maximal final ethanol level (17–18%) was obtained with only minimal residual sugar left [17]. A further increase in glucose concentration above this level reduced the maximal ethanol level again. Cells were first pre-grown in 3 mL of YPD medium for 24 h (200 rpm, 30°C), after which 0.5 mL was transferred to 5 mL of YP+5% (w/v) glucose and the culture incubated for 24 h (200 rpm, 30°C). Cells of the last pre-culture were inoculated in 100 mL of YP+10% (w/v) glucose with initial OD600 of 1.0. The cells were grown for 2 days (200 rpm, 30°C) until stationary phase. 12.5×10^9^ cells, based on cell counting, were harvested. The cells were centrifuged (3000 rpm, 5 min, 4°C), the pellet was resuspended in 3 mL of YP and inoculated into 250 mL of YP+33% (semi-static) or 35% (continuous stirring) (w/v) glucose. The fermentations were performed at 25°C. Agitation was performed with a magnetic rod (30×6 mm) at 120 rpm (semi-static, 4 h) or 200 rpm (continuous stirring). The fermentation was followed by weighing the tubes and from the weight loss the glucose leftover was calculated. Samples were taken at the end of the fermentation for HPLC analysis and cell viability determination. The metabolites quantified by HPLC were glucose, glycerol and acetic acid. The HPLC system utilized (Waters Breeze) consisted of an ion-exclusion column (WAT010290) at 75°C and detection was performed by refractive index (model 2414). The eluent used was H_2_SO_4_ (5 mM) at a flow rate of 1.0 mL/min. Samples of 10 µL were automatically injected and processed for 20 min. Ethanol was quantified by near infrared spectroscopy (Alcolyzer, Anton Paar). Cell viability was assessed by oxonol staining followed by flow cytometry analysis [42]. The ethanol yield (g of ethanol produced per g of glucose consumed) was calculated by dividing the ethanol produced with the glucose consumed (initial glucose concentration minus glucose leftover).

### Ethanol tolerance assays of cell proliferation on solid media

The cells were pre-grown in YPD for 2 days (200 rpm, 30°C). The OD600 was measured in triplicate and the cells were diluted to an initial OD600 of 0.5. Four serial dilutions were made (10^−1^, 10^−2^, 10^−3^ and 10^−4^). A volume of 4 µL was spotted on plates: YPD (control), YPD+16% (v/v) ethanol, YP+16% (v/v) ethanol, YPD+18% (v/v) ethanol, YP+18% (v/v) ethanol and YPD+20% (v/v) ethanol. The plates were incubated at 30°C for up to 11 days and growth was scored from the second day on. The ethanol levels indicated are initial ethanol levels. During the preparation and incubation of the plates some ethanol may evaporate. Therefore, sample and control strains were always put together on the same plates.

### Sporulation and tetrad dissection

General procedures for sporulation and tetrad dissection were used [43].

### Determination of mating type

A small amount of cells (1.5 mg) was incubated with 10 µL of NaOH (0.02N) for 1 h (RT). The determination of the mating type was done by PCR with the primers for the MAT locus and *MAT* ***a*** and *MATα* (alpha) DNA [44]. The 3 primers were used together.

### Genomic DNA extraction and whole-genome sequence analysis

Preparation of the DNA pools from the segregants was done either by (1) individual genomic DNA extraction and pooling of the DNA in equimolar concentrations; (2) mixing of the cells, based on dry weight, prior to DNA extraction, or (3) mixing of the cells based on OD600, prior to DNA extraction. For all preparations, the genomic DNA was extracted according to Johnston [45]. At least 3 µg of DNA per pool was provided for whole-genome sequencing to both GATC Biotech GA (Konstanz, Germany) and Beijing Genomics Institute (BGI, Hong Kong, China). In both cases the sequencing was performed with the Illumina platform and gave for most of the genome, and especially in the QTL areas, very similar results. For both pools and at both companies the sequencing depth was ∼38 and the read length was 75 at GATC Biotech and 90 at BGI.

### Bioinformatics analysis and confirmation of QTLs

Assembly and mapping were done with DNAstar Lasergene software. Smoothing of the sequencing data was performed with a Linearized Mixed Model (LMM) framework [5], [22]. We implemented a Hidden Markov Model (HMM) to identify regions related with the phenotypes similar to the one implemented in the FastPHASE package [25]. For each variant, the HMM has three possible states: (i) relation with the superior parent, (ii) relation with the control parent and (iii) no relation (background). To capture the effect of recombination, the transition between two states of the same type is the probability of no recombination and the probability of the transition between two states of different type is the probability of recombination divided by two. We estimated the probability of recombination for each pair of neighbor variants using a negative exponential relation with the physical distance as in [25]. The emission of each state is the number of calls of the alternative allele which is an integer between zero and n_i_, where n_i_ is the total number of allele calls for the variant i. We used beta-binomial distributions for all states to take into account the fact that given the finite number of segregants, the contribution of each parent to the pool is not exactly half. For the superior parent states we setup α = 10 and β = 1. For the control parent states we set α = 1 and β = 10. For the background states we estimated α and β using the alternative allele frequencies in all sites. We checked that for the background distribution α≈β>1, which makes the background distribution to be close to a binomial with probability 0.5 (as expected). We used the forward-backward algorithm to calculate the posterior probability of each state given the allele counts for each dataset. A manuscript with a complete explanation of the algorithm and comparisons with currently available methods is in preparation. The QTLs detected were further analyzed by scoring SNPs in the segregants individually using allele-specific primer sets, which were rigorously tested for reliability with the two variants of each SNP in the parent strains and all segregants. Statistically significant QTLs were confirmed by multiple testing using a false discovery rate (FDR) control [46].

### Molecular Biology methods

Yeast cells were transformed with the LiAc/SS-DNA/PEG method [47]. Genomic DNA was extracted with PCI [phenol/chlroform/isoamyl-alcohol (25∶24∶1)] [48]. Polymerase chain reaction (PCR) was performed with Accuprime polymerase (Invitrogen) for sequencing purposes and ExTaq (Takara) for diagnostic purposes. Sanger sequencing was performed by the Genetic Service Facility of the VIB. The detection of SNPs by PCR was performed as previously described [5].

### Reciprocal hemizygosity analysis (RHA)

RHA was performed as described previously [5], [10] in the diploid Seg5/BY710 genetic background. In addition to single gene deletions we also performed large deletions (bulk RHA) of regions up to 27 kb long. The selection marker utilized was the amidase gene (*AMD1*), which was amplified from the vector pF6a-AMD1-MX6. The gene *AMD1* was cloned from *Z. rouxii* [49]. The primers utilized in the *AMD1* amplification had at least 80 extra bases that corresponded to the flanking regions of the area to be deleted. The transformants were selected on solid YCB + acetamide 10 mM (yeast carbon base 11.7 g/L; sodium phosphate buffer 0.03 M; agar 20 g/L). The correct integration of the constructs was checked by PCR, using one primer that annealed within *AMD1* and two other primers that annealed either downstream or upstream of the deleted region. The PCR products were sequenced and the polymorphisms (SNPs and indels) present in the regions flanking the selection marker were identified when the Seg5 allele was replaced by *AMD1*. On the other hand, when the laboratory allele was deleted, no polymorphism was detected by Sanger sequencing. Double allele deletion was not observed during the bulk RHA because the deleted regions contained at least one essential gene.

### Reproducibility and statistical analysis

The fermentations with different yeast strains were done with the reference strain V1116 as a control in duplicate. The most interesting strains were repeated at least once. The fermentations with different meiotic segregants were done with the reference strains Seg5, BY710 and Seg5/BY710. The segregants showing more than 16.5% (v/v) ethanol production were evaluated by fermentation at least once more. The fermentations for RHA were done in triplicate. The results were analyzed with a paired t-test (p<0.01, except for the comparison of V1116 and CBS1585 for which p<0.05 was used).

### Data access

All sequence data have been deposited in the Sequence Read Archive (SRA) at the National Center for Biotechnology Information (NCBI) and can be accessed with account number SRA056812.

## Supporting Information

Table S1 Maximal ethanol accumulation capacity and ethanol tolerance of cell proliferation. Screening of 68 yeast strains in small-scale fermentations for maximal ethanol accumulation (250 mL YP+33% glucose). Ethanol production is shown in comparison to the robust wine strain V1116 and the strains are listed in descending order of performance. The final ethanol titer (%, v/v), glycerol level (g/L) and ethanol yield (%) are also indicated for each strain. The strains were either evaluated once, twice (*), three times (**) or six times (***). †Ethanol yield is expressed as percentage of the maximum theoretical ethanol yield (0.51 g ethanol/g glucose consumed). Ethanol tolerance of cell proliferation was measured in YPD agar plates with the indicated concentrations of ethanol. The indicated score is the number of dilutions in which the strains grew (maximum = 4).
(DOC)

Table S2 *Saccharomyces cerevisiae* strains utilized in this study.
(DOC)
