# Supplementary material for: Comparative Polygenic Analysis of Maximal Ethanol Accumulation Capacity and Tolerance to High Ethanol Levels of Cell Proliferation in Yeast
Source: PLoS Genet. 2013 Jun 6;9(6):e1003548. doi: 10.1371/journal.pgen.1003548 (PMC3675000; doi:10.1371/journal.pgen.1003548)
Supplement: Table S1 — Maximal ethanol accumulation capacity and ethanol tolerance of cell proliferation. Screening of 68 yeast strains in small-scale fermentations for maximal ethanol accumulation (250 mL YP+33% glucose). Ethanol production is shown in comparison to the robust wine strain V1116 and the strains are listed in descending order of performance. The final ethanol titer (%, v/v), glycerol level (g/L) and ethanol yield (%) are also indicated for each strain. The strains were either evaluated once, twice (*), three times (**) or six times (***). †Ethanol yield is expressed as percentage of the maximum theoretical ethanol yield (0.51 g ethanol/g glucose consumed). Ethanol tolerance of cell proliferation was measured in YPD agar plates with the indicated concentrations of ethanol. The indicated score is the number of dilutions in which the strains grew (maximum = 4). (DOC) [file pgen.1003548.s001.doc]

**Supplementary table 1**

| **Strains** | **Relative ethanol production (% compared to V1116)** | **EtOH % (v/v)** | **Glycerol (g/L)** | **Ethanol yield (%)†** | **Ethanol tolerance of cell proliferation (10 days growth)** | | | | |
| --- | --- | --- | --- | --- | --- | --- | --- | --- | --- |
|  |  |  |  |  | **12%** | **14%** | **16%** | **18%** | **20%** |
| CBS1585 (***) | 103.4 | 18.8 | 10.9 | 88.4 | 4 | 4 | 4 | 3 | 1 |
| Benvinda (*) | 102.0 | 18.6 | 11.6 | 88.1 | 4 | 4 | 4 | 4 | 2 |
| Ethanol Red (**) | 101.9 | 18.5 | 13.1 | 87.7 | 4 | 4 | 3 | 1 | 0 |
| Eau de Vie (**) | 101.7 | 18.4 | 10.4 | 88.3 | 4 | 4 | 4 | 4 | 4 |
| Fermivin (**) | 101.7 | 18.8 | 11.2 | 88.0 | 4 | 4 | 3 | 1 | 0 |
| Produtor 4 | 101.6 | 17.8 | 11.7 | 88.1 | 3 | 2 | 2 | 2 | 2 |
| Alcotec 24 (*) | 101.5 | 18.8 | 11.9 | 88.0 | 4 | 4 | 0 | 0 | 0 |
| Alcotec 48 (*) | 101.5 | 18.8 | 12.0 | 87.8 | 4 | 4 | 0 | 0 | 0 |
| Alcotec 23% (*) | 101.5 | 18.8 | 12.2 | 87.6 | 4 | 4 | 0 | 0 | 0 |
| Alcotec vodka star (*) | 101.5 | 18.8 | 12.2 | 87.7 | 4 | 4 | 0 | 0 | 0 |
| Turbo yeast (*) | 101.5 | 18.8 | 12.5 | 87.7 | 4 | 3 | 2 | 0 | 0 |
| Intek796 (*) | 101.2 | 18.8 | 12.6 | 87.4 | 4 | 3 | 4 | 4 | 0 |
| Thermosacc Dry (*) | 99.9 | 17.2 | 9.8 | 88.5 | 4 | 3 | 2 | 0 | 0 |
| CBS7961 | 99.2 | 17.0 | 10.8 | 88.4 | 3 | 3 | 3 | 4 | 0 |
| Alcotec triple (*) | 98.9 | 18.2 | 12.6 | 87.5 | 4 | 4 | 4 | 3 | 0 |
| Zimmerman 814 | 98.9 | 18.5 | 11.5 | 87.9 | 4 | 4 | 4 | 4 | 4 |
| Montanhesa Atividade | 98.9 | 17.4 | 11.9 | 87.8 | 4 | 4 | 4 | 2 | 2 |
| TMB3399 | 98.6 | 18.9 | 10.5 | 88.4 | 4 | 4 | 4 | 4 | 3 |
| CAT1 (*) | 97.8 | 17.5 | 11.3 | 88.1 | 4 | 4 | 4 | 4 | 4 |
| Fali S1 | 97.8 | 18.0 | 12.7 | 87.4 | 4 | 4 | 0 | 0 | 0 |
| CBS6414 | 97.3 | 16.7 | 10.7 | 88.3 | 0 | 0 | 0 | 0 | 0 |
| CBS7957 | 97.2 | 18.3 | 13.5 | 87.1 | 4 | 3 | 3 | 3 | 2 |
| Sake 4134 | 96.3 | 18.6 | 14.5 | 86.8 | 3 | 3 | 3 | 4 | 0 |
| VR1 (*) | 96.1 | 17.2 | 10.7 | 88.3 | 4 | 4 | 4 | 4 | 4 |
| PE2 (*) | 96.1 | 17.2 | 11.6 | 88.0 | 4 | 4 | 4 | 4 | 4 |
| CBS7960 | 96.0 | 16.8 | 10.5 | 88.2 | 3 | 4 | 4 | 4 | 2 |
| Diva | 96.0 | 16.9 | 9.9 | 88.5 | 4 | 4 | 3 | 2 | 1 |
| Montanhesa Pé | 94.9 | 17.8 | 13.1 | 87.2 | 4 | 4 | 4 | 3 | 2 |
| M2 | 94.7 | 17.8 | 11.1 | 87.9 | 3 | 3 | 3 | 3 | 1 |
| French Red | 93.9 | 17.6 | 7.5 | 89.3 | 4 | 4 | 4 | 4 | 0 |
| Superstart (*) | 93.7 | 17.0 | 11.6 | 88.0 | 1 | 2 | 2 | 2 | 0 |
| CBS2808 | 93.5 | 16.1 | 10.5 | 88.2 | 3 | 3 | 3 | 2 | 2 |
| Produtor 3 | 93.4 | 16.5 | 10.9 | 88.2 | 4 | 4 | 4 | 4 | 2 |
| Sake K11 | 93.3 | 17.1 | 12.8 | 87.6 | 4 | 4 | 4 | 3 | 1 |
| Sauternes | 93.3 | 17.6 | 11.5 | 88.0 | 3 | 3 | 3 | 2 | 1 |
| CBS6413 | 93.1 | 16.0 | 11.1 | 88.0 | 4 | 4 | 4 | 4 | 4 |
| CBS6412 (*) | 92.9 | 16.9 | 7.2 | 89.8 | 3 | 2 | 3 | 2 | 0 |
| Champagne | 92.5 | 17.4 | 11.8 | 87.8 | 4 | 3 | 3 | 3 | 2 |
| Zimermman 815 | 92.4 | 17.8 | 11.0 | 87.9 | 4 | 4 | 4 | 4 | 3 |
| *S. boulardii* | 92.4 | 16.3 | 10.6 | 88.2 | 4 | 4 | 4 | 2 | 0 |
| CBS1198 | 92.2 | 17.4 | 9.8 | 88.7 | 2 | 0 | 1 | 0 | 0 |
| CBS7764 | 91.9 | 17.3 | 10.5 | 88.6 | 3 | 4 | 3 | 1 | 0 |
| Fali S2 | 91.3 | 17.2 | 12.1 | 87.9 | 3 | 1 | 0 | 0 | 0 |
| TMB3400 | 91.3 | 17.6 | 10.6 | 88.4 | 4 | 4 | 4 | 4 | 4 |
| Cognac | 90.1 | 17.4 | 12.0 | 87.8 | 4 | 4 | 4 | 4 | 1 |
| 46EDV (*) | 89.3 | 16.8 | 9.2 | 89.1 | 3 | 3 | 3 | 2 | 0 |
| CBS2807 | 88.9 | 15.3 | 11.2 | 88.1 | 3 | 2 | 3 | 3 | 0 |
| CBS1252 | 87.9 | 16.6 | 12.7 | 87.5 | 4 | 4 | 3 | 2 | 0 |
| CBS7072 | 87.5 | 16.5 | 11.1 | 88.3 | 4 | 4 | 4 | 4 | 3 |
| CBS7958 | 86.1 | 16.1 | 11.5 | 88.1 | 4 | 4 | 4 | 4 | 4 |
| CBS1390 | 86.0 | 16.1 | 9.3 | 89.3 | 4 | 4 | 4 | 2 | 1 |
| Pasteur Champagne | 85.3 | 16.0 | 8.7 | 89.4 | 4 | 3 | 3 | 3 | 0 |
| Port | 83.4 | 15.7 | 10.3 | 88.5 | 4 | 3 | 4 | 3 | 1 |
| Y55 | 82.6 | 15.0 | 9.5 | 88.9 | 4 | 4 | 4 | 4 | 3 |
| S288c (*) | 81.2 | 14.9 | 10.8 | 88.6 | 4 | 4 | 4 | 3 | 0 |
| Assmanhausen | 79.7 | 15.0 | 9.5 | 89.0 | 4 | 4 | 4 | 4 | 1 |
| CBS7539 | 78.2 | 14.7 | 11.2 | 88.1 | 4 | 4 | 4 | 4 | 1 |
| CBS1200 | 76.5 | 14.3 | 8.7 | 89.2 | 4 | 4 | 4 | 3 | 0 |
| Westmalle | 76.0 | 14.1 | 8.8 | 89.3 | 0 | 0 | 0 | 0 | 0 |
| CBS1241 | 74.8 | 14.1 | 9.7 | 89.0 | 4 | 4 | 1 | 2 | 0 |
| CBS382 | 74.7 | 14.1 | 10.8 | 88.4 | 4 | 3 | 4 | 4 | 1 |
| GT344 (*) | 69.0 | 13.4 | 8.8 | 89.4 | 2 | 0 | 0 | 0 | 0 |
| GT339 (*) | 68.7 | 13.3 | 9.2 | 89.2 | 2 | 0 | 0 | 0 | 0 |
| GT336 (*) | 67.1 | 13.0 | 9.1 | 89.2 | 2 | 0 | 0 | 0 | 0 |
| CMBS33 (*) | 66.0 | 12.5 | 10.0 | 88.7 | 2 | 2 | 1 | 0 | 0 |
| BY4741 (*) | 64.3 | 12.1 | 9.7 | 89.1 | 4 | 4 | 3 | 0 | 0 |
| CBS422 | 62.0 | 11.7 | 13.9 | 87.2 | 3 | 3 | 2 | 2 | 3 |
| CBS436 | 60.8 | 10.4 | 11.6 | 88.2 | 4 | 4 | 4 | 3 | 0 |
