# Supplementary material for: Comparative Polygenic Analysis of Maximal Ethanol Accumulation Capacity and Tolerance to High Ethanol Levels of Cell Proliferation in Yeast
Source: PLoS Genet. 2013 Jun 6;9(6):e1003548. doi: 10.1371/journal.pgen.1003548 (PMC3675000; doi:10.1371/journal.pgen.1003548)
Supplement: Table S2 — Saccharomyces cerevisiae strains utilized in this study. (DOC) [file pgen.1003548.s002.doc]

**Supplementary table 2**

| **Strain** | **Description/use** | **Reference/origin** |
| --- | --- | --- |
| BY4741 | *Mat****a*** *his3∆1 leu2∆0 ura3∆0 met15∆0* | (Brachmann et al. 1998) |
| BY4742 | *Matα his3∆1 leu2∆0 ura3∆0 lys2∆0* | (Brachmann et al. 1998) |
| BY4743 | *Mat****a****/α his/his leu/leu ura/ura met/MET LYS/lys* | (Brachmann et al. 1998) |
| S288c | *Mat****a*** prototroph | (Brachmann et al. 1998) |
| BY710 | BY4742 derivative; *Matα his3∆1 leu2∆0 ura3∆0 lys2∆0* | Lab stock |
| CBS1585 | Heterothallic diploid sake strain with high ethanol production capacity | Centraalbureau voor Schimmelcultures, Utrecht, The Netherlands |
| Seg5 | Haploid derived from CBS1585 with the same phenotype, *Mat****a*** | This study |
| Seg5xBY710 | Diploid obtained by crossing Seg5 with pAMS710 | This study |
| V1116 | Homothallic diploid wine strain | Lallemand, Canada |
| CAT1 | Brazilian bioethanol production | Fermentec, Brazil |
| VR1 | Brazilian bioethanol production | Fermentec, Brazil |
| PE2 | Brazilian bioethanol production | Fermentec, Brazil |
| CBS1198 | Sake | Centraalbureau voor Schimmelcultures, Utrecht, The Netherlands |
| CBS436 | Sake | Centraalbureau voor Schimmelcultures, Utrecht, The Netherlands |
| CBS6412 | Sake (Kyokai n°7) | Centraalbureau voor Schimmelcultures, Utrecht, The Netherlands |
| CBS6413 | Sake (Kyokai n°5) | Centraalbureau voor Schimmelcultures, Utrecht, The Netherlands |
| CBS6414 | Sake | Centraalbureau voor Schimmelcultures, Utrecht, The Netherlands |
| CBS7539 | Beer, Bulgaria | Centraalbureau voor Schimmelcultures, Utrecht, The Netherlands |
| CBS382 | Beer, Brazil | Centraalbureau voor Schimmelcultures, Utrecht, The Netherlands |
| CBS422 | Beer, Ukraine | Centraalbureau voor Schimmelcultures, Utrecht, The Netherlands |
| CMBS33 | Lager beer strain | Centre for malting and brewing collection, KULeuven |
| GT336 | CMBS33 variant | (Blieck et al. 2007) |
| GT339 | CMBS33 variant | (Blieck et al. 2007) |
| GT344 | CMBS33 variant | (Blieck et al. 2007) |
| Westmalle | Beer bottle yeast isolate | Isolated from Westmalle triple beer (9.5% v/v alcohol) |
| CBS1252 | *S. cerevisiae* or *S. paradoxus* | Centraalbureau voor Schimmelcultures, Utrecht, The Netherlands |
| CBS1390 | Wine, Hungary | Centraalbureau voor Schimmelcultures, Utrecht, The Netherlands |
| CBS7764 | *Salmo gairducrii* (rainbow trout), Sweden | Centraalbureau voor Schimmelcultures, Utrecht, The Netherlands |
| CBS7957 | Factory of cassava flour, Brazil | Centraalbureau voor Schimmelcultures, Utrecht, The Netherlands |
| CBS7958 | Factory of cassava flour, Brazil | Centraalbureau voor Schimmelcultures, Utrecht, The Netherlands |
| CBS1241 | *S. cerevisiae* or *S. paradoxus* | Centraalbureau voor Schimmelcultures, Utrecht, The Netherlands |
| Produtor 3 | Cachaça (spirit) production | Sugar cane fermentation, UFOP, Brazil |
| Produtor 4 | Cachaça (spirit) production | Sugar cane fermentation, UFOP, Brazil |
| Montanhesa Atividade | Cachaça (spirit) production | Sugar cane fermentation, UFOP, Brazil |
| Diva | Cachaça (spirit) production | Sugar cane fermentation, UFOP, Brazil |
| Benvinda | Cachaça (spirit) production | Sugar cane fermentation, UFOP, Brazil |
| Montanhesa Pé | Cachaça (spirit) production | Sugar cane fermentation, UFOP, Brazil |
| CBS7959 | Bioethanol from sugar cane | Brazil |
| CBS7960 | Bioethanol from sugar cane | Brazil |
| CBS7961 | Bioethanol from sugar cane | Brazil |
| 46EDV | Bioethanol | Lallemand, Canada |
| Thermosacc Dry | Bioethanol | Lallemand, Canada |
| Superstart | Bioethanol | Lallemand, Canada |
| Ethanol Red | Bioethanol | Lesaffre, France |
| Fali S1 | Bioethanol | AB Mauri, Australia |
| Fali S2 | Bioethanol | AB Mauri, Australia |
| *S. boulardii* | Probiotic | Enterol 250 mg (Biodiphar) |
| Y55 | Prototroph diploid | Lesaffre Development, France |
| Sake4134 | Sake | Homebrewers warehouse |
| TMB3399 | Xylose utilization | (Wahlbom et al. 2003) |
| TMB3400 | Xylose utilization | (Wahlbom et al. 2003) |
| CBS1200 | *S. cerevisiae* or *S. paradoxus* | Centraalbureau voor Schimmelcultures, Utrecht, The Netherlands |
| Alcotec 24h | Bioethanol | Alcotec, United Kingdom |
| Alcotec 48h | Bioethanol | Alcotec, United Kingdom |
| Alcotec 23% | Bioethanol | Alcotec, United Kingdom |
| Turbo yeast | Bioethanol | Alcotec, United Kingdom |
| Vodka star | Spirit | Alcotec, United Kingdom |
| Turbo triple still | Spirit | Alcotec, United Kingdom |
| CBS2807 | Wine (Slovakia) | Centraalbureau voor Schimmelcultures, Utrecht, The Netherlands |
| CBS2808 | Wine (Slovakia) | Centraalbureau voor Schimmelcultures, Utrecht, The Netherlands |
| CBS7072 | Bioethanol | Centraalbureau voor Schimmelcultures, Utrecht, The Netherlands |
| Eau de vie | Spirit | WYEAST Laboratories |
| French Red | Wine | UCDavis, USA |
| Riesling Hefe | Homothallic diploid | Zimmermann F. (Darmstadt) |
| SIHA3 | Homothallic diploid | Zimmermann F. (Darmstadt) |
| Pasteur Champagne | Wine | UCDavis, USA |
| Intek796 | Wine | UCDavis, USA |
| Fermivin | Wine | Oenobrands, France |
| M2 | Wine | UCDavis, USA |
| Sauternes | Wine | UCDavis, USA |
| Champagne | Wine | UCDavis, USA |
| Port | Spirit | UCDavis, USA |
| Cognac | Spirit | UCDavis, USA |
| Sake K11 | Sake | National Research Institute of Brewing, Japan |

References:

Blieck L, Toye G, Dumortier F, Verstrepen KJ, Delvaux FR, Thevelein JM, Van Dijck P (2007) Isolation and characterization of brewer's yeast variants with improved fermentation performance under high-gravity conditions. Appl Environ Microbiol 73: 815-824.

Brachmann CB, Davies A, Cost GJ, Caputo E, Li J, Hieter P, Boeke JD (1998) Designer deletion strains derived from *Saccharomyces cerevisiae* S288C: a useful set of strains and plasmids for PCR-mediated gene disruption and other applications. Yeast 14: 115-132.

Wahlbom CF, van Zyl WH, Jonsson LJ, Hahn-Hagerdal B, Otero RR (2003) Generation of the improved recombinant xylose-utilizing *Saccharomyces cerevisiae* TMB 3400 by random mutagenesis and physiological comparison with Pichia stipitis CBS 6054. *FEMS Yeast Res* 3: 319-326.
